# Antibody-based CCR5 blockade protects Macaques from mucosal SHIV transmission

**DOI:** 10.1038/s41467-021-23697-6

**Published:** 2021-06-07

**Authors:** Xiao L. Chang, Gabriela M. Webb, Helen L. Wu, Justin M. Greene, Shaheed Abdulhaqq, Katherine B. Bateman, Jason S. Reed, Cleiton Pessoa, Whitney C. Weber, Nicholas Maier, Glen M. Chew, Roxanne M. Gilbride, Lina Gao, Rebecca Agnor, Travis Giobbi, Jeffrey Torgerson, Don Siess, Nicole Burnett, Miranda Fischer, Oriene Shiel, Cassandra Moats, Bruce Patterson, Kush Dhody, Scott Kelly, Nader Pourhassan, Diogo M. Magnani, Jeremy Smedley, Benjamin N. Bimber, Nancy L. Haigwood, Scott G. Hansen, Timothy R. Brown, Lishomwa C. Ndhlovu, Jonah B. Sacha

**Affiliations:** 1 Vaccine & Gene Therapy Institute, Portland, OR USA; 2 Oregon National Primate Research Center, Oregon Health & Science University, Portland, OR USA; 3 University of Hawaii, Honolulu, HI USA; 4 IncellDX, Menlo Park, CA USA; 5 Amarex Clinical Research LLC, Germantown, MD USA; 6 CytoDyn Inc., Vancouver, WA USA; 7 MassBiologics of the University of Massachusetts Medical School, Boston, MA USA; 8 Palm Springs, Palm Springs, CA USA; 9 Department of Medicine, Division of Infectious Disease, Weill Cornell Medicine, New York, NY USA

**Keywords:** Antibody therapy, HIV infections

## Abstract

In the absence of a prophylactic vaccine, the use of antiretroviral therapy (ART) as pre-exposure prophylaxis (PrEP) to prevent HIV acquisition by uninfected individuals is a promising approach to slowing the epidemic, but its efficacy is hampered by incomplete patient adherence and ART-resistant variants. Here, we report that competitive inhibition of HIV Env-CCR5 binding via the CCR5-specific antibody Leronlimab protects rhesus macaques against infection following repeated intrarectal challenges of CCR5-tropic SHIV_SF162P3_. Injection of Leronlimab weekly at 10 mg/kg provides significant but partial protection, while biweekly 50 mg/kg provides complete protection from SHIV acquisition. Tissue biopsies from protected macaques post challenge show complete CCR5 receptor occupancy and an absence of viral nucleic acids. After Leronlimab washout, protected macaques remain aviremic, and adoptive transfer of hematologic cells into naïve macaques does not transmit viral infection. These data identify CCR5 blockade with Leronlimab as a promising approach to HIV prophylaxis and support initiation of clinical trials.

## Abstract (web summary)

CCR5 is a co-receptor for many transmitted HIV strains. Here, the authors show that biweekly injection of the CCR5-specific antibody Leronlimab protects rhesus macaques against infection following repeated intrarectal challenges of a CCR5-tropic SHIV.

## Introduction

Pre-exposure prophylaxis (PrEP) is effective for HIV prevention, where drug concentrations in the blood are strongly correlated with protection^1,2^. However, the efficacy of PrEP is hindered by incomplete drug adherence^3^ and the global rising rate for antiretroviral therapy (ART) drug resistance^4^, resulting in incidents of multidrug-resistant HIV infections despite high adherence to PrEP^5,6^. Potent broadly neutralizing antibodies (bNAbs) represent a potential alternative to ART-based PrEP. Yet, bNAbs also remain susceptible to antibody-resistant HIV strains, and alternate preventative modalities with complementary mechanisms of action are needed^7–10^.

HIV can utilize either the CCR5 or CXCR4 co-receptor for entry into CD4+ T cells, yet CCR5 is the primary co-receptor used during transmission of HIV^11–13^. Accordingly, individuals with a natural genetic deficiency in CCR5 via a homozygous 32-base pair deletion in *ccr5* (CCR5^Δ32/Δ32^) are highly resistant to HIV infection^14,15^. Further underscoring the central role of CCR5 in viral spread in vivo, the only two documented cases of HIV cure occurred in the setting of allogenic stem cell transplantation using CCR5^Δ32/Δ32^ donor cells^16,17^. CCR5 therefore represents an ideal target for HIV prevention, yet small-molecule CCR5 inhibitors like Maraviroc have yielded disappointing results as PrEP agents^18,19^ and alternate CCR5-specific strategies are needed.

Leronlimab is an anti-CCR5 humanized IgG4 antibody currently in clinical trials for HIV therapy as a once weekly, subcutaneous injection with a favorable safety profile in over 1000 volunteers across multiple studies^20^. In contrast to Maraviroc, which interferes with HIV Env attachment to CCR5 by allosteric modulation, Leronlimab binds to the same CCR5 extracellular loop-2 and N-terminus domains used by HIV Env, thereby directly outcompeting HIV for binding to CCR5 (ref. ^21^). A single 10 mg/kg dose of Leronlimab lowered plasma viral loads by approximately 100-fold for 2 weeks in HIV-positive individuals^22–24^. When utilized as once weekly self-administered subcutaneous monotherapy, Leronlimab maintained undetectable plasma viral loads in HIV-infected individuals for over 2 years^25^, with the longest successful monotherapy patients now reaching over 6 years of continual use (Chang et al., manuscript in preparation). The ability to self-administer Leronlimab at home as a subcutaneous injection augurs well for its adherence profile as a PrEP agent. Finally, in both single dose and multiyear monotherapy studies, no viral co-receptor switching occurred, underscoring the high genetic barrier to developing Leronlimab resistance. Based on these antiviral, safety, and user-friendly characteristics, we hypothesized that Leronlimab could be utilized as an effective PrEP strategy with the potential for high patient usage and set out to establish the efficacy of Leronlimab-based PrEP in the macaque model of HIV.

## Results

**Fig. 1:**
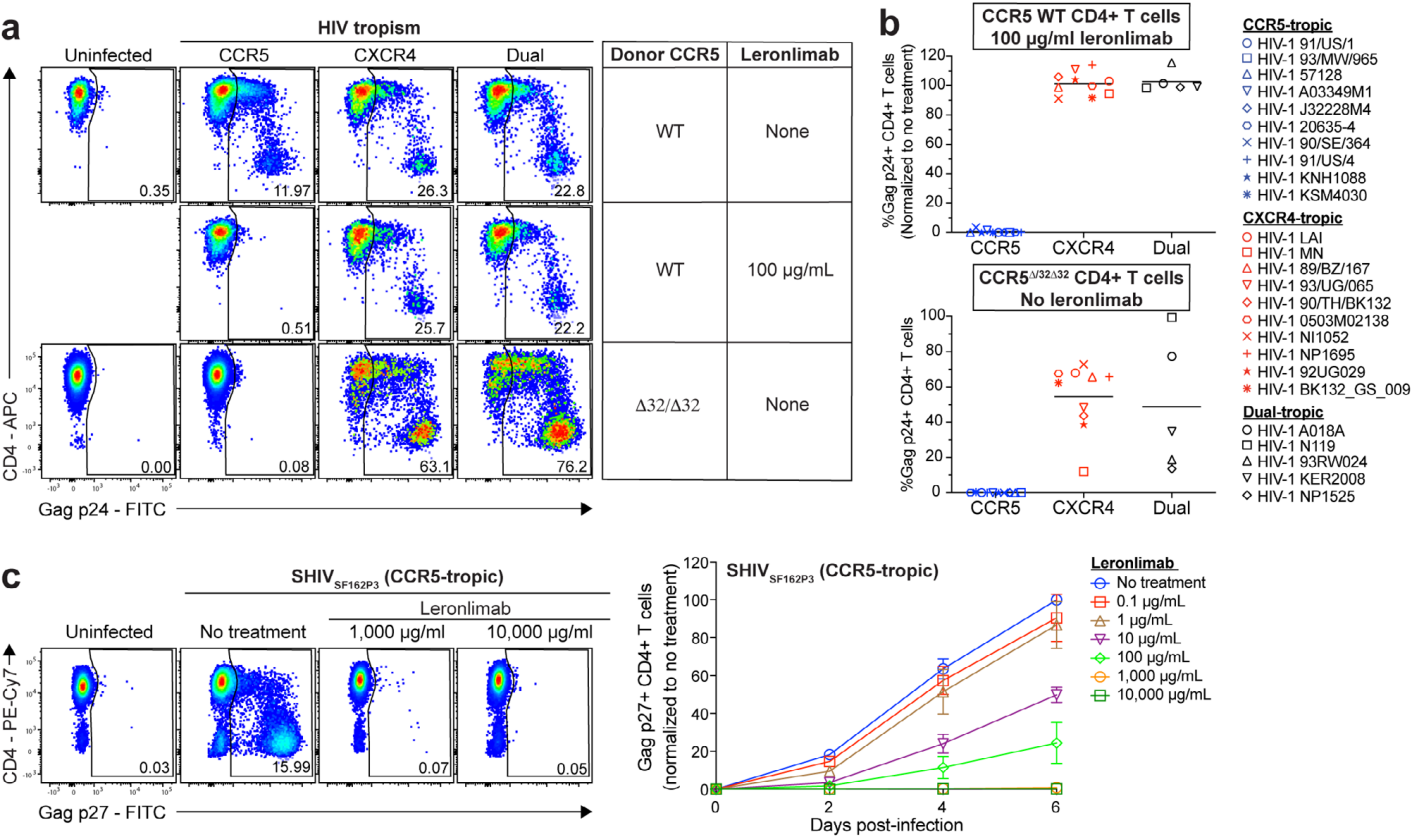
Leronlimab blocks the spreading of CCR5-utilizing strains in vitro. **a** Representative flow cytometry plots displaying intracellular Gag p24 staining of untreated CCR5 wild type (WT), Leronlimab-treated CCR5 WT, and untreated CCR5^Δ32/Δ32^ human CD4+ T cells in HIV-1 infection assay with CCR5-tropic (blue), CXCR4-tropic (red), and dual-tropic viruses (black). **b** Summary of the mean Gag p24 levels from HIV-1 spreading assays performed on CCR5 WT human CD4+ T cells treated with 100 μg/mL Leronlimab (top) or untreated CCR5^Δ32/Δ32^ human CD4+ T cells (bottom). Each data point represents the mean Gag p24 levels of CCR5 WT human (*n* = 3) or CCR5^Δ32/Δ32^ human (*n* = 1; two replicates) CD4+ T cells. Each viral tropism group represents the mean Gag p24 levels of ten CCR5-tropic, ten CXCR5-tropic, and five dual-tropic viral infections. Gag p24 levels of CCR5 WT human CD4+ T cells were normalized to “no treatment”. **c** Spreading assay using SHIV_SF162P3_, a CCR5-utilizing virus. Macaque CD4+ T cells (*n* = 3) were infected and treated with increasing amounts of Leronlimab. (Right) Summary (mean ± SEM) of the longitudinal infection as measured by Gag p27 levels, with all values normalized to day 6 “no treatment”. Source data are provided as a Source Data file.

Because cell-associated virus plays an important role in mucosal transmission^26^, we first assessed the ability of Leronlimab to inhibit HIV cell-to-cell transmission in an in vitro spreading assay. In neutralization assays, Leronlimab inhibits diverse HIV isolates with an IC_50_ value comparable to HIV bNAbs such as 10E8, PGT128, and 3BNC117 (refs. ^27,28^). However, in line with previous findings of reduced neutralizing activity on cell-to-cell transmission^29^, 100 μg/mL Leronlimab was necessary to fully prevent cell-to-cell spread of CCR5-tropic HIV in the in vitro culture system of purified, highly activated CD4+ T cells (Supplementary Fig. 1). When treated with this concentration of Leronlimab in the spreading assay, CD4+ T cell targets from CCR5 wild-type donors became fully resistant to infection with CCR5-tropic HIV while still supporting replication of CXCR4- and dual-tropic HIV, identical to the infection pattern observed with CD4+ T cell targets from a CCR5^Δ32/Δ32^ donor (Fig. 1a). Using a panel of 25 HIV isolates from multiple clades (Supplementary Table 1), we confirmed the ability of Leronlimab to mimic the resistance conferred by the CCR5^Δ32/Δ32^ phenotype onto CD4+ T cells from CCR5 wild-type donors, thereby protecting these cells from infection with CCR5-tropic isolates from multiple geographical origins (Fig. 1b).

CCR5 is highly conserved between humans and rhesus macaques^30^, and accordingly, Leronlimab specifically bound CCR5 on the surface of macaque CD4+ T cells (Supplementary Fig. 2a, b). However, while the frequency of CCR5+ CD4+ T cells was similar between human and macaques in peripheral blood, macaque CD4+ T cells expressed a higher number of CCR5 molecules on a per-cell basis, with central memory CD4+ T cells expressing the highest levels overall (Supplementary Fig. 2c). This higher per-cell expression of CCR5 on macaque CD4+ T cells suggested that inhibition of SHIV infection in macaque CD4+ T cells may require higher concentrations of CCR5-targeted competitive inhibitors such as Leronlimab compared to inhibition of HIV infection in human CD4+ T cells. Indeed, a 10-fold higher concentration of Leronlimab was required to achieve full inhibition of SHIV_SF162P3_ infection in macaque cells compared to HIV in human cells in vitro (Fig. 1c and Supplementary Fig. 1a).

**Fig. 2:**
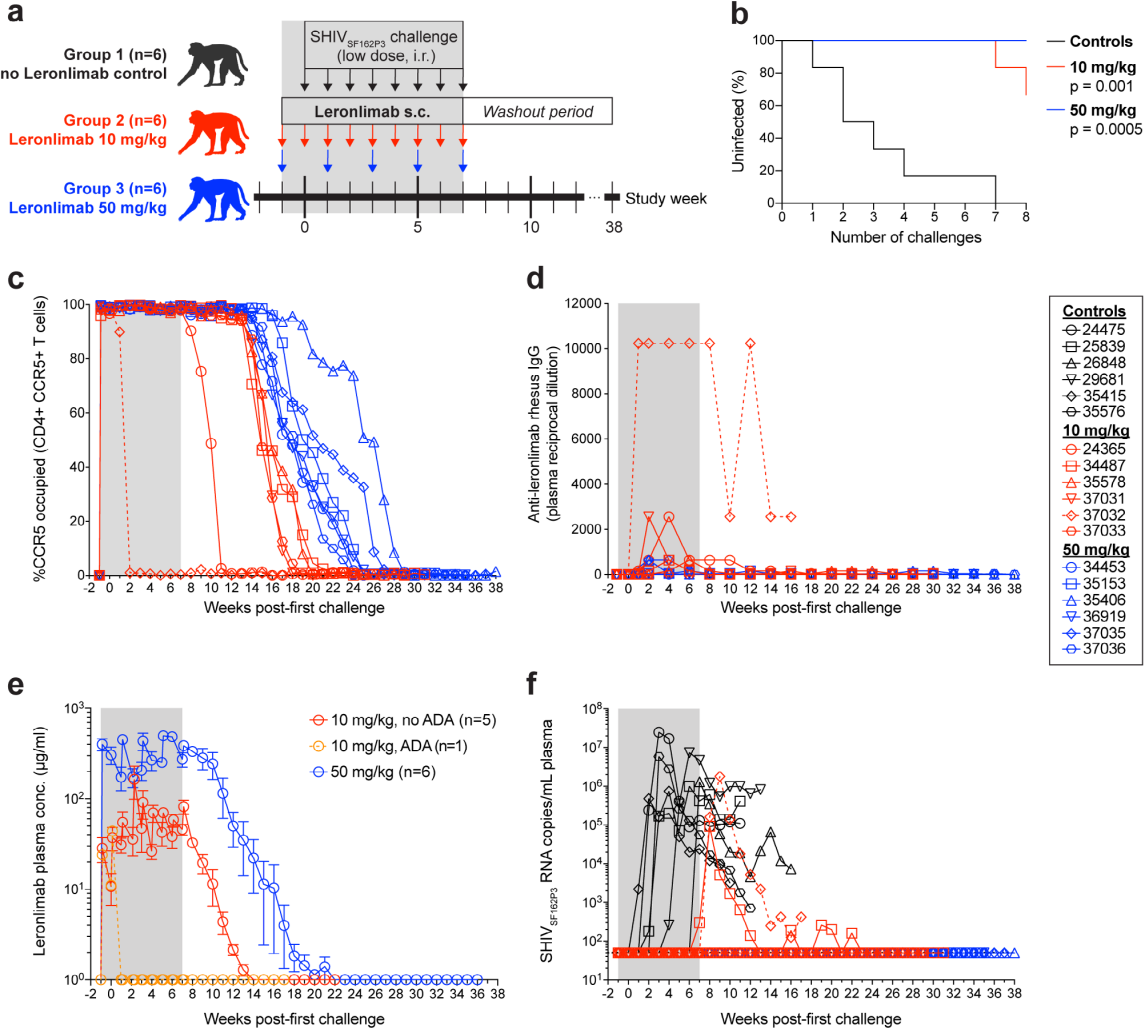
Leronlimab pre-exposure prophylaxis protects rhesus macaques from intrarectal SHIVSF162P3 acquisition. **a** Study outline. Eighteen rhesus macaques were challenged with 3.2 TCID_50_ of SHIV_SF162P3_ weekly via intrarectal inoculation for 8 consecutive weeks. Group 1 received no Leronlimab (black, *n* = 6) while Group 2 received weekly 10 mg/kg Leronlimab (red, *n* = 6) and Group 3 received biweekly or once-every-two-weeks Leronlimab (blue, *n* = 6). **b** Kaplan–Meier curve comparing the percentage of uninfected to the number of SHIV_SF162P3_ challenges, analyzed by log-rank test with Dunn’s correction. **c** Longitudinal CCR5 receptor occupancy levels on peripheral blood CCR5+ CD4+ T cells in Leronlimab-treated macaques as determined by flow cytometry (see “Methods”). **d** Longitudinal anti-Leronlimab rhesus IgG levels in plasma of Leronlimab-treated macaques. **e** Mean (±SEM) longitudinal Leronlimab plasma concentrations in Groups 2 and 3, separated by development of persistent anti-Leronlimab rhesus IgG antibody levels (ADA antidrug antibodies; orange). **f** Longitudinal SHIV_SF162P3_ plasma viral loads. Horizontal dashed line denotes assay limit of quantification (50 copies/mL); gray boxes in **a**, **c**–**f** denote the Leronlimab treatment phase for Leronlimab-treated macaques. Legend at right shows symbols used in panels **c**, **d**, **f** identifying individual rhesus macaques. The macaque that developed persistent anti-Leronlimab rhesus IgG antibody levels (37032) is shown as a dashed line throughout panels **c**–**f**. Group colors and individual animal symbols are consistent throughout the manuscript. Source data are provided as a Source Data file.

The primary co-receptor used during mucosal transmission is CCR5 (refs. ^11–13^). Thus, we explored whether Leronlimab could act as PrEP to protect macaques from repeated low-dose intrarectal (IR) SHIV_SF162P3_ challenges. The study design, shown in Fig. 2a, consisted of three groups of macaques (*n* = 6 per group, individual macaque information listed in Supplementary Table 2) that served as untreated controls (group 1), weekly 10 mg/kg Leronlimab-treated (group 2), or biweekly 50 mg/kg Leronlimab-treated animals (group 3). The 10 mg/kg dose in macaques is an allometrically scaled human clinical dose of 350 mg Leronlimab. Due to the emergence of antidrug antibodies (ADA), the allometrically scaled human clinical dose of 700 mg Leronlimab was adjusted to 50 mg/kg dose in macaques, a dose that does not elicit ADA likely due to high zone tolerance^31^, with dosing changed to biweekly to more closely approximate plasma levels from weekly doses in humans. Thus, these two macaque doses of Leronlimab represent the lowest and highest doses currently used in clinical trials. Animals received their first subcutaneous Leronlimab injection one week prior to the start of IR challenges. At study week zero, all animals received their first IR challenge with 3.2 TCID_50_ of SHIV_SF162P3_ that continued for eight consecutive weeks. We selected SHIV_SF162P3_ for the following two reasons: (1) the parental Env from HIV-1_SF162_ is CCR5-tropic, yet particularly resistant to CCR5-targeting agents^27^ and (2) SHIV_SF162P3_ can switch to CXCR4 co-receptor use in macaques via defined amino acid substitutions in Env V3 loop^32^. Uninfected animals received their last IR challenge and Leronlimab injection at study week 7. After this point, infected animals were euthanized at 10 weeks after confirmed infection while aviremic, protected animals were longitudinally monitored and euthanized 8 weeks after loss of Leronlimab receptor occupancy (RO) on peripheral blood CD4+ T cells.

In all animals, Leronlimab was well-tolerated and showed no adverse clinical effects, similar to the high safety profile reported previously in multiple clinical trials^20,22–25^. Serum chemistry and complete blood count parameters did not differ significantly between groups nor change significantly over the study period (Supplementary Fig. 3). Although no significant change in peripheral blood T cell counts or frequencies were found, we observed a dose-dependent increase in peripheral blood CCR5+ T cell frequencies and absolute counts during Leronlimab treatment that subsequently returned to baseline levels concomitant with Leronlimab washout, likely reflecting the previously described ability of Leronlimab to interfere with CCR5-mediated chemotaxis^21^ (Supplementary Fig. 4).

**Fig. 3:**
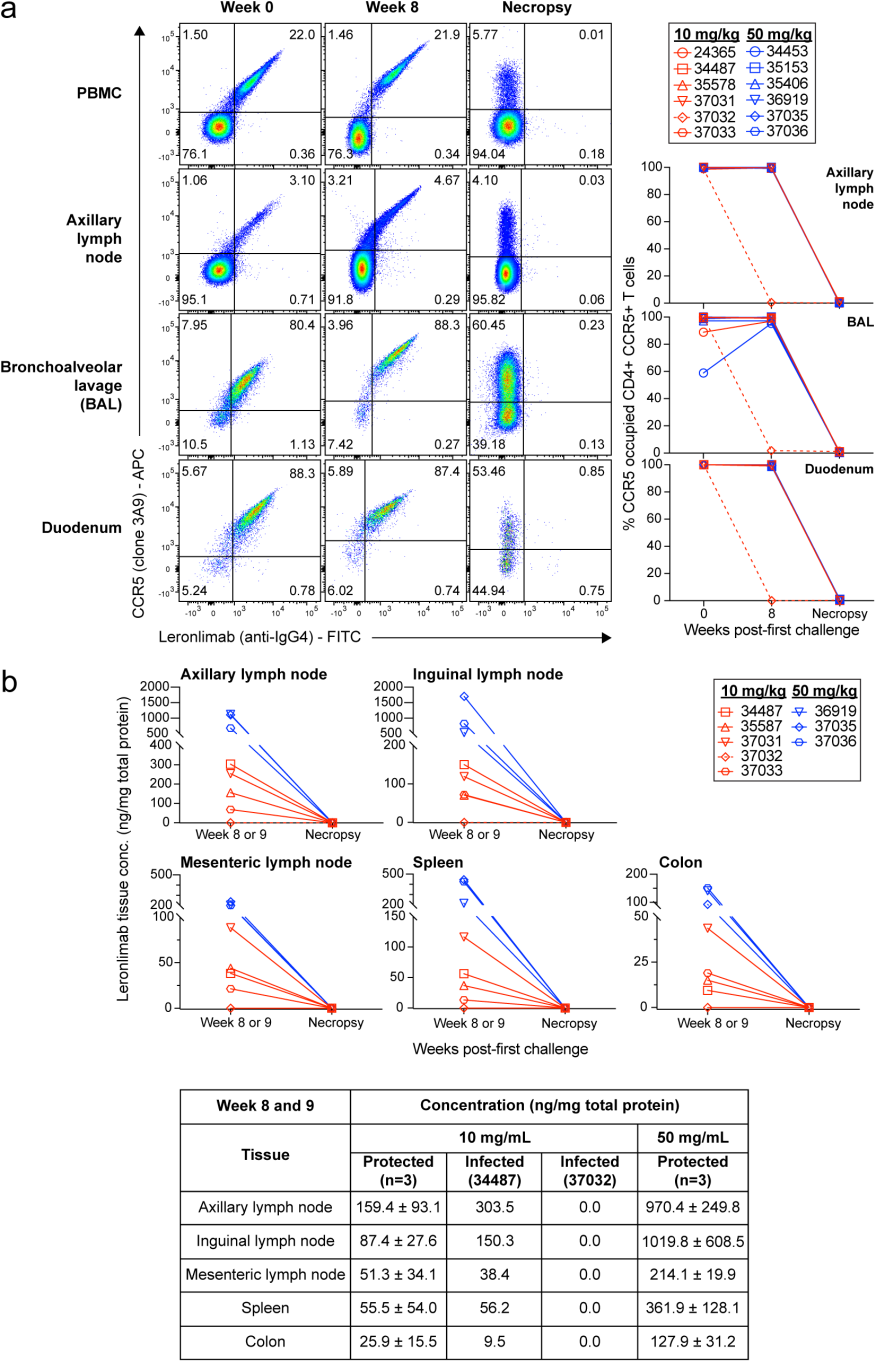
Longitudinal Leronlimab tissue levels and CCR5 receptor occupancy. Showing 10 mg/kg treated animals (red) and 50 mg/kg treated animals (blue). **a** On the left, representative flow cytometry plots showing the co-staining of anti-CCR5 (clone 3A9) and Leronlimab (by anti-human IgG4, clone HP-6025) on CD4+ T cells from PBMC, axillary lymph node, bronchoalveolar lavage, and duodenum. Tissues were taken from three timepoints: week 0 (one-week post first Leronlimab treatment; week of first viral challenge), week 8 (one-week post final Leronlimab treatment; one-week post final viral challenge), and necropsy (varying for all animals). Flow cytometry plots for all three timepoints and tissues followed the 50 mg/kg treated animal, 35153. On the right, individual CCR5 RO on longitudinal CD4+ CCR5+ T cells from axillary lymph node, bronchoalveolar lavage, and duodenum. Dashed line shows the CCR5 RO of 37032, the animal that developed ADA. **b** Tissue concentration of Leronlimab for five 10 mg/kg treated animals and three 50 mg/kg treated animals during two timepoints: week 8/9 and necropsy. On the bottom, summary of the tissue concentration in mean ( ± SD) values for the three protected 10 mg/kg treated animals; individual values for the infected 10 mg/kg treated animals, 34487, and 37032; and mean ( ± SD) values for the three protected 50 mg/kg treated animals. Group colors and individual animal symbols are consistent throughout the manuscript. Source data are provided as a Source Data file.

All untreated control macaques in group 1 became infected by the seventh IR challenge, while two of six macaques in group 2 (weekly 10 mg/kg) became infected (*p* = 0.001). In contrast, no macaques in group 3 (50 mg/kg biweekly) became infected (*p* = 0.0005), indicating that Leronlimab protected macaques from SHIV acquisition in a dose-dependent manner (Fig. 2b). To track CCR5 RO, we established and validated an ex vivo Leronlimab RO assay (Supplementary Fig. 5). All six macaques in group 3 maintained full CCR5 RO on peripheral blood CD4+ T cells throughout the challenge phase (Fig. 2c). As expected with the high dose, these macaques did not develop ADA (Fig. 2d), and maintained sufficient plasma levels with a half-life of 7.5 weeks after the last Leronlimab injection at study week 7 (Fig. 2e). Following washout of plasma Leronlimab and loss of CCR5 RO on peripheral blood CD4+ T cells, we monitored animals for an additional 8 weeks for emergence of occult SHIV infection. All group 3 macaques remained aviremic until necropsy, suggesting sterilizing protection from acquisition (Fig. 2f). In the four protected group 2 animals (weekly 10 mg/kg), we observed earlier loss of CCR5 RO following the final Leronlimab dose, low level ADA development, and, similar to all group 3 animals, no emergence of occult SHIV infection following Leronlimab washout and loss of CCR5 RO, again indicating sterile protection from acquisition (Fig. 2c–f). Two group 2 animals, 37032 and 34487, however, did become infected. 37032 developed strong ADA that led to rapid loss of CCR5 RO, clearance of plasma Leronlimab in plasma, and acquisition of SHIV (Fig. 2c–f). 34487 acquired infection despite maintaining peripheral blood CD4+ T cell CCR5 RO and Leronlimab plasma levels similar to group 2 protected animals. Interestingly, following the final challenge this animal had the lowest Leronlimab levels in the colon of any treated animals without ADA (Fig. 3b), suggestive of incomplete CCR5 RO in the rectum that resulted in suboptimal protection during viral challenges. Following infection, 34487 experienced the lowest peak plasma viremia of all infected animals and was the only animal to control SHIV_SF162P3_ to undetectable levels, suggesting antiviral effects by Leronlimab similarly seen in clinical trials of monotherapy treatment of HIV-positive individuals^20,22–25^ (Fig. 2f and Supplementary Fig. 6). However, we cannot exclude the potential of spontaneous control of SHIV replication. We sequenced the V3 loop of *env* and found that virions in the plasma remained CCR5-tropic for both animals, excluding the possibility of infection by CXCR4-tropic SHIV variants (Supplementary Fig. 7).

To determine if protection correlated with CCR5 RO on tissue and mucosal resident CD4+ T cells, we collected lymph node and endoscopic duodenum biopsies and bronchoalveolar lavages (BAL) during the first IR challenge and again 1 week following the final IR SHIV challenge. We observed full CCR5 RO on CD4+ T cells from these tissues throughout the IR challenge phase, except for animal 37032 who lost CCR5 RO due to ADA (Fig. 3a). To assess Leronlimab tissue penetration, we biopsied various anatomical locations following the final SHIV challenge. As expected, higher levels of Leronlimab were present in group 3 versus group 2 animals, with the lowest colon levels found in 34487 (Fig. 3b). Finally, we examined tissues collected during necropsy and confirmed an absence of Leronlimab and CCR5 RO on CD4+ T cells from these tissues, demonstrating that the lack of plasma viremia in protected animals was not due to residual Leronlimab in tissue.

**Fig. 4:**
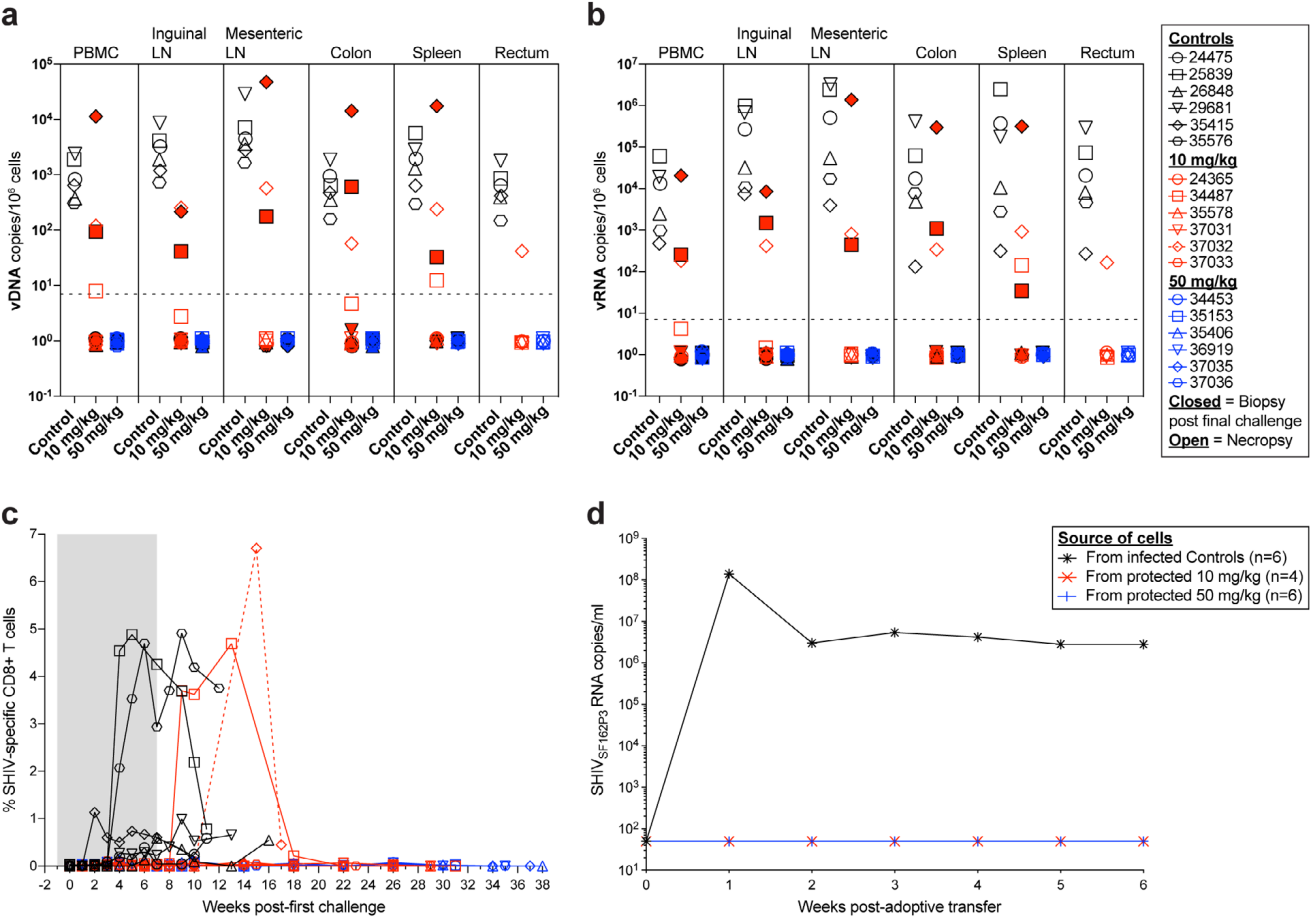
Validation of protected Leronlimab-treated rhesus macaques. Showing untreated control animals (black), 10 mg/kg treated animals (red), and 50 mg/kg treated animals (blue). **a**, **b** Cell-associated SHIV_SF162P3_ DNA (a) and RNA (b) viral loads after final challenge (closed symbols, biopsies at study week 8 or 9) and at necropsy (open symbols). Horizontal dashed lines in **a** and **b** denote assay limit of quantification of 7 copies/10^6^ cells. **c** Longitudinal SHIV-specific CD8+ T cell responses as determined by intracellular cytokine staining. Positive percentage of responses were determined by Boolean gating to CD8+ T cells that were CD69+/TFN-α+ and/or CD69+/IFN-γ+. Gray box denotes the Leronlimab treatment phase for Leronlimab-treated macaques. **d** Longitudinal SHIV_SF162P3_ plasma viral loads in adoptive transfer recipients infused with cells harvested from macaques of each group upon euthanasia (one recipient per group). Each adoptive transfer recipient received a combined infusion of cells from six infected control macaques, from four aviremic 10 mg/kg treated macaques, or from six aviremic 50 mg/kg treated macaques (see Supplementary Table 3 for breakdown of infused cells). Horizontal dashed line denotes assay limit of quantification (50 copies/mL). Group colors and individual animal symbols are consistent throughout the manuscript. Source data are provided as a Source Data file.

To assess if Leronlimab mediated sterile protection from challenge, we measured cell-associated SHIV DNA and RNA levels in multiple anatomical locations by tissue biopsy after the final challenge (study weeks 8–9) and at necropsy (Fig. 4a, b). As expected, SHIV nucleic acid was readily detected in macaques with plasma viremia, including in all untreated controls and the two infected group 2 animals, 34487 and 37032. However, many tissues from 34487 at necropsy were below the limit of quantification suggesting Leronlimab may have limited viral spread post acquisition. In contrast, we found no samples positive for SHIV DNA or RNA from the four aviremic group 2 animals and all six group 3 animals. Because Gag- and Vif-specific CD8+ T cells are a sensitive readout of occult SIV infection^33^, we longitudinally monitored for their emergence in all macaques. In line with longitudinal plasma and cell-associated viral load results (Figs. 2f and 4a, b), SHIV Gag- and Vif-specific CD8+ T cells responses developed in all viremic animals while it was absent in all aviremic animals, despite the presence of CMV-specific CD8+ T cells in all macaques (Fig. 4c and Supplementary Fig. 8). Finally, after confirming that Leronlimab and CCR5 RO was not present in tissues collected at necropsy (Fig. 3), we adoptively transferred pooled hematologic cells into one SHIV-naïve recipient macaque per group. Lymph node (axillary, inguinal, and mesenteric), bone marrow, and spleen cells from all six infected group 1 animals, the four protected group 2 animals, and all six group 3 animals were pooled by group and infused at final cell counts of 7.8 × 10^8^, 6.9 × 10^8^, and 9.7 × 10^8^ cells, respectively (Supplementary Table 3). Transfer of SHIV_SF162P3_ infection occurred with the infusion of cells from infected control animals, but not isolated cells from protected animals in group 2 or 3 (Fig. 4d). Together, these results indicate that Leronlimab mediated sterile protection against mucosal acquisition of SHIV_SF162P3_ in the aviremic animals.

## Discussion

The efficiency of daily ART-based PrEP is heavily dependent on patient adherence^3^ and absence of drug-resistance variants^6^. Even new preventative approaches like long-acting injectable cabotegravir can lead to the emergence of drug-resistant variants, especially if initiated during undiagnosed acute infection^34^. Thus, alternative and cost-reducing methods are needed, including the use of antibody-based PrEP. Leronlimab is administered subcutaneously at home, offering advantages over bNAb-based PrEP that requires IV infusion and intra-muscular injectables that are administered in a clinic by a health-care provider. Given the significantly longer Leronlimab plasma half-life in humans versus macaques, coupled with the lower per-cell CCR5 expression on CD4+ T cells in humans, it is possible that once monthly Leronlimab may be sufficient to prevent HIV acquisition. With advances in antibody Fc engineering to enhance plasma half-life^35^, minor sequence modifications could extend Leronlimab to a once-quarterly injection to further improve adherence by lowering the frequency of administration.

Because CCR5 is the primary HIV co-receptor used by virions during transmission^11–13^, development of viral resistance is difficult, as demonstrated by the previously documented protection from infection in CCR5^Δ32/Δ32^ individuals^14,15^. Indeed, no escape mutants were observed with long-term Leronlimab monotherapy use in HIV+ individuals, in contrast to treatment with bNAbs^8,9^. This is likely due to targeting of the primary CCR5 co-receptor rather than epitopes on Env by bNAbs that permit selection for bNAb-resistance variants. Infection with CXCR4- or dual-tropic HIV isolates is possible, but such events represent a small minority of transmission due to multiple layers of restriction on CXCR4-tropic viruses in the mucosa^9,35^. Further, the ability of Leronlimab to fully protect animals from rectal transmission of SHIV is in contrast to a previous study where the small-molecule CCR5-inhibitor Maraviroc failed to protect animals challenged with 10 TCID_50_ SHIV_SF162P3_ despite sufficiently high drug concentrations in rectal tissues^18^. Thus, competitive inhibition of the CCR5 Env interaction represents a powerful approach for PrEP. Finally, beyond targeting CCR5 for prophylaxis, significant effort is focused on genetic engineering approaches to knock out CCR5 in HIV cure strategies^36^. The results presented here show that the use of Leronlimab could mimic the same protection from acquisition seen in CCR5^Δ32/Δ32^ individuals via a reversible pharmacological therapy that does not require genetic modifications that risk off-target effects. Given that the CCR5^Δ32/Δ32^ phenotype carries no risk for life span^37^ and that multiyear Leronlimab use has been safely demonstrated, future studies are warranted to explore the utility of Leronlimab in HIV cure and prevention.

## Methods

### HIV and SHIV stocks

HIV-1 isolates were obtained from the NIH AIDS Reagent Program, with the majority from the HIV-1 60 International Isolate Panel (Cat #11412). The SHIV_SF162P3_ stock (harvest 4, dated 9/16/2016, 173.3 ng/mL p27, 1 × 10^9^ vRNA copies/mL, 2.67 × 10^5^ TCID_50_/mL in TZM-bl cells, 1.28 × 10^3^ TCID_50_/mL in rhesus PBMC (peripheral blood mononuclear cells)) used in the PrEP animal study was generated, characterized, and kindly provided by Nancy Miller.

### HIV and SHIV in vitro infection assays

Human PBMC were first depleted of CD8+ T cells with Human CD8 Microbeads (Miltenyi) and then sequentially enriched for CD4+ T cells with Human CD4 Microbeads (Miltenyi) following the manufacturer’s instructions. The resulting CD4+ CD8− T cells were incubated at 2 × 10^6^ cells/mL in R15-100 media (RPMI 1640 with antibiotic/mycotic, 15% fetal bovine serum (FBS), and 100 U/mL IL-2) and activated for 24 h with a stimulating cocktail containing CD3, CD49d, CD28 antibodies (BD Biosciences), and Staphylococcal enterotoxin B (Toxin Technologies, Inc.). After 24 h, cells were washed two times and incubated for 2–3 additional days in R15-100 before viral infection. At day 0 of infection, 5 × 10^5^ cells were incubated with or without the desired concentration of Leronlimab for 1 h at 37 °C. Next, cells were infected with the desired HIV isolate from Supplementary Table S1 by spinoculation for 2 h at 1200 × *g* at room temperature (RT). Cells were washed four times with R15-100 to remove free viruses and cultured with R15-100 media plus the same concentration of Leronlimab used during the pre-treatment step. An additional 5 × 10^5^ cells were left uninfected and kept in culture as the uninfected control. Cultures were maintained by replacing 50% of the culture with new media containing the desired concentration of Leronlimab every other day for 5 days, when cells were harvested for intracellular p24 staining by flow cytometry.

Rhesus macaque (RM) PBMC were depleted of CD8+ T cells by staining with NHP CD8-PE (Miltenyi) followed by anti-PE microbeads (Miltenyi) then subsequently enriched for CD4+ T cells with NHP CD4 microbeads (Miltenyi), following the manufacturer’s instructions. Purified RM CD4+ cells were activated and maintained in culture similarly to human CD4+ T cells, as described above. For the spreading assay with HIV-1 Ba-L, HIV-1 LAI, and SHIV_SF162P3_, CD4+ T cells were isolated, activated, infected, and cultured as described above but with the following changes. HIV-1 Ba-L, HIV-1 LAI, and SHIV_SF162P3_ were used to infect at a multiplicity of infection (MOI) of 1 × 10^−5^ (FFU/cell) and kept in culture for 6 days before p24 or p27 intracellular staining. For detection of intracellular HIV p24 or SHIV p27 by flow cytometry, cells were stained for CD3, CD4, CD8, and amine-reactive dye for viability for 30 min at RT in the dark. Cells were washed once with phosphate-buffered saline (PBS), spun down at 830 × *g* for 4 min, and fixed with 2% paraformaldehyde (PFA) for 30 min in 4 °C. Afterwards, cells were washed once with FACS buffer (10% bovine growth serum in PBS) and stained with p24 or p27 antibodies in 100 μL of Permeabilization Medium B (Thermo Fisher) for 1 h at RT in the dark. Cells were washed once with FACS buffer and fixed again with 2% PFA for more than 30 min before collecting on LSR-II instrument and FACsDIVA version 6.1 (BD Biosciences, Franklin Lakes, NJ). Data were analyzed using FlowJo v10 (Tree Star) by gating on singlet, live, CD3+, CD8−, and p24+ or p27+.

### Human blood donors

Healthy human donor whole blood was purchased from Innovative Research and processed in-house to PBMC by density gradient centrifugation using Ficoll-Hypague. Blood was collected with K2 EDTA anticoagulant and tested negative for the following viral markers: HIV-1 RNA, antibodies to HIV, antibodies to hepatitis C virus (HCV), HCV RNA, hepatitis B virus (HBV) DNA, hepatitis B surface antigen (HbsAg), and syphilis. CCR5 expression was confirmed via flow cytometry. Leukapheresis samples were collected from TRB following informed consent under the Research and Institutional Review Committee (RIRC) of the Queens Medical Center approval number RA-2014-307 of the H026 Study protocol.

Individual consented to identification in this study.

### Quantitation of CCR5 expression levels

To measure the frequency of CCR5-expressing cells, PBMCs were incubated with 5 μg/mL of unlabeled Leronlimab for 30 min at RT in the dark, and then washed once with PBS. Anti-human IgG4 was used as a secondary antibody to detect surface-bound Leronlimab for 30 min at RT in the dark. Cells were washed once with FACS buffer and once with PBS, and then stained for CD3, CD4, CD8, CCR5 (via antibody clone 3A9, which is specific to a distinct, non-competitive CCR5 epitope than Leronlimab), and amine-reactive dye for 30 min at RT in the dark. Cells were washed twice with PBS and fixed with 2% PFA before collecting through the LSR-II instrument and FACsDIVA version 6.1. Samples were analyzed by gating on singlet, live, CD3+, CD4+/CD8−, and CCR5+ (via clone 3A9) and/or human IgG4+ events. The number of CCR5 molecules on the cell surface was measured with quantitative cytometry using the Quantum Molecules of Equivalent Soluble Fluorochrome (MESF) kit (Bangs Laboratories, Inc). PE-conjugated Leronlimab used to quantify surface CCR5 expression and PE-labeled microspheres for standard curve generation were provided by IncellDX. Phenotypic staining was done using CD3−, CD4−, CD8−, CD14−, CD16− specific antibodies used as described above. T cell memory subset determination was defined as central memory (human: CCR7+ CD45RA−, RM: CD28+ CD95+), effector memory (human: CCR7− CD45RA−, RM: CD28− CD95+), and naive (human: CCR7+ CD45RA+, RM: CD28+ CD95−). Gating was done using FlowJo v10. MFI was used to attain MESF according to the manufacturer’s protocol.

### Leronlimab CCR5 RO

To measure the percentage of CCR5 RO on the surface of CD4+ T cells, we developed the RO equation shown in Supplementary Fig. 5.

$$\% {\rm{RO}}=\tfrac{ \% {\rm{IgG}}4}{ \% {\rm{IgG}}4+ \% {\rm{Leronlimab}}-{\rm{PB}}}{\times}100 \%$$

The equation measures unoccupied CCR5 receptors by using Pacific Blue-conjugated Leronlimab (termed Leronlimab-PB). CCR5 RO is defined as the percentage of cells CCR5+ (measured by clone 3A9) and Leronlimab+ (measured by anti-human IgG4) divided by the percentage of cells CCR5+ and Leronlimab+ (measured by the sum of anti-human IgG4 and Leronlimab-PB) cells following incubation with a saturating concentration of Leronlimab-PB. This method is based on RO assays for anti-PD-1 antibodies in clinical trials^38^.

PBMC or single cells from tissue homogenates (0.3–1 × 10^6^) were stained with anti-human IgG4 for 30 min at RT in the dark. Next, cells were washed once with FACS buffer and three times with PBS and then stained with CD45, CD3, CD4, CD8, CD16, CD14, amine-reactive dye, CCR5 (via antibody clone 3A9), and Leronlimab-PB for 30 min at RT in the dark. Finally, cells were washed twice with PBS and fixed with 2% PFA for more than 30 min before collecting on LSR-II instrument and FACsDIVA version 6.1. Using FlowJo v10, cells were gated on CD45+, singlet, live, CD3+, CD4+, and CCR5+ (via 3A9 staining) events. The CD4+ CCR5+ population was further gated on human IgG4+ or Leronlimab-PB+ events.

### Rhesus macaques

All study RM were housed at the Oregon National Primate Research Center (ONPRC) in ABSL-2+ rooms with autonomously controlled temperature, humidity, and lighting. At assignment, all study RM were free of cercopithicine herpesvirus 1, D-type simian retrovirus, simian T-lymphotrophic virus type 1, and *Mycobacterium tuberculosis*. RM were typed for the MHC alleles Mamu-A*01, Mamu-A*02, Mamu-B*17, and Mamu-B*08, with Mamu-B*17 and/or -B*08 positive animals excluded when possible or placed into control groups when not possible to exclude biasing results. All attempts were made to pair housed RM during the study period. When RM were single cage-housed due to infection status, they had visual, auditory, and olfactory contact with other animals, and an enhanced enrichment plan was designed and overseen by RM behavior specialists. RM were fed commercially prepared primate chow twice daily and received supplemental fresh fruit or vegetables daily. Fresh, potable water was provided via automatic water systems. RM were sedated with ketamine HCl or dexmedetomidine for procedures, including subcutaneous Leronlimab administration, venipuncture, BAL, tissue biopsy, and SHIV challenge. For spleen and mesenteric lymph node biopsies, animals were sedated with isoflurane. At scheduled endpoints, RM were euthanized with sodium pentobarbital overdose (>50 mg/kg) and exsanguinated via the distal aorta, and tissue collection at necropsy was performed by a certified veterinary pathologist. RM care and all experimental protocols and procedures were approved by the ONPRC Institutional Animal Care and Use Committee (IACUC). The ONPRC is a Category I facility. The Laboratory Animal Care and Use Program at the ONPRC is fully accredited by the American Association for Accreditation of Laboratory Animal Care (AAALAC) and has an approved Assurance (#A3304-01) for the care and use of animals on file with the NIH Office of Laboratory Animal Welfare. The IACUC adheres to national guidelines established in the Animal Welfare Act (7 U.S.C. Sections 2131–2159) and the Guide for the Care and Use of Laboratory Animals (8th Edition) as mandated by the U.S. Public Health Service Policy.

For the Leronlimab PrEP study, a total of 18 adult RMs were used and divided between three experimental groups: (1) six RMs in the control group, (2) six RMs in the 10 mg/kg weekly Leronlimab-treated group, and (3) six RMs in the 50 mg/kg every 2 weeks Leronlimab-treated group. The three groups were gender matched, with two females and four male RMs in each group. See Table S3 for the gender, age, and MHC type of these animals. Animals received their first injection of Leronlimab 1 week before the initiation of viral challenges, and continued to receive Leronlimab until study week 7, for a total of nine Leronlimab injections for group 1 (10 mg/kg treated group) and a total of five Leronlimab injections for group 2 (50 mg/kg treated group). At study week 0, all three groups received their first IR SHIV_SF162P3_ challenge with a 1:400 dilution (3.2 TCID_50_ measured from rhesus PBMC, dose based on in vivo macaque titration experiments, Nancy Miller, personal communication) delivered via atraumatic installation into the rectum with a needleless syringe. Viral challenge then continued every week until confirmed infection or until study week seven for a total of eight consecutive weekly IR challenges. Animals were euthanized as described above, with the timing dictated by the following guidelines: (1) 10 weeks after acquisition of SHIV_SF162P3_ infection, which was confirmed by plasma viremia, or (2) 8 weeks after complete washout of plasma Leronlimab and loss of Leronlimab CCR5 RO on CD4+ T cells in blood. Three SHIV-naïve RMs served as adoptive transfer recipients for tissue homogenates from PrEP RM as described. All Leronlimab utilized in these studies was clinical-grade material provided by CytoDyn at a concentration of 175 mg/mL.

### Processing of blood and tissue

Whole blood was collected into EDTA-treated or non-anticoagulant tubes (BD Biosciences). Blood in EDTA-tubes was assessed for complete blood counts using an ABX Pentra 60C+ Hematology Analyzer. Blood in non-anticoagulant tubes was spun down at 1860 × *g* for 10 min to separate serum from clotted blood, and then assessed for serum chemistry values using an ABX Pentra 400 Chemistry Analyzer. PBMC and plasma were isolated from whole blood in EDTA-treated tubes by density gradient centrifugation using Ficoll-Hypaque. Bronchoalveolar lavage fluid was collected in PBS and filtered through 70-μm cell strainers. Lymph node and spleen were collected in RPMI 1640 containing 10% FBS (R10), diced into tiny pieces with a scalpel, and forced through 70-μm cell strainers to collect single-cell suspensions. Bone marrow was collected in R10, pelleted by spinning at 830 × *g* for 4 min. Cell pellet was resuspended by vigorously shaking in PBS containing 2 nM EDTA. Cell suspension was spun down again at 830 × *g* for 4 min and resuspended in 70% isotonic Percoll (GE Healthcare, Buckinghamshire, UK) before underlaying in 37% isotonic Percoll. Cells were spun at 500 × *g* for 20 min with brake. Mononuclear cells in the center interface were collected and washed in R10. Duodenum and colon were collected in R10 and diced into tiny pieces with a scalpel. Tissues were placed into a 50 mL conical tube containing R3 (RPMI 1640 and 3% FBS) with 0.5 M EDTA, and then incubated in 37 °C with 225 r.p.m. shaking for 30 min to remove mucus coating. After 30 min, cells were poured over a tea strainer and sequentially washed three times with Hank’s Buffered Salt Solution (HBSS) to remove EDTA and mucus coating. Tissues were returned into a 50 mL conical tube containing R3, 0.2 mg/mL collagenase (Sigma-Aldrich, St. Louis, MO), and 0.2 mg/mL DNase I (Roche, Indianapolis, IN), and then incubated in 37 °C with 225 r.p.m. shaking for 1 h. Digested tissues were filtered through a 70-μm cell strainers. Cell flow throughs were spun at 840 × *g* for 4 min. Pellet was resuspended in 70% isotonic Percoll and underlaid in 37% isotonic Percoll, before spinning at 500 × *g* for 20 min with brake. Mononuclear cells in the center interface were collected and washed in R10.

To quantify Leronlimab concentration and ADA in the blood, plasma was heat inactivated by incubation at 56 °C for 30 min, and then spun for 20 min at 12,000 × *g* to pellet residual debris. Resulting supernatant was transferred to a new tube and stored at −80 °C until assayed. To quantify Leronlimab concentration in tissues, tissues were finely diced and 10–1000 mg of tissue of interest were placed into Lysing Matrix tubes (MP Biomedicals) and 300–500 μL of complete EDTA-free Protease Inhibitor Cocktail (Sigma-Aldrich) in PBS was added. Tissue disruption was achieved by beating in a Beadbeater (Biospec) device for three cycles of 1 min beating and 1 min on ice. Supernatant from the tissue homogenate was transferred to a new tube and spun for 20 min at 12,000 × *g* to pellet residual debris. Resulting supernatant was transferred to a new tube and stored at −80 °C until assayed.

### Viral nucleic acid detection

Nucleic acid from plasma and PBMC cell pellets were extracted using the Maxwell 16 instrument (Promega, Madison, WI) following the manufacturer’s protocol, which uses the LEV Viral Nucleic Acid Kit for plasma and the LEV Whole Blood Nucleic Acid kit for cell pellets.

Nucleic acid from tissues were extracted by first placing tissues in Lysing Matrix tubes (MP Biomedicals) with 1 mL Tri-reagent (Molecular Research Center, TR-118). Then, vortexed to soaked tissues in Tri-reagent and placed on wet ice. Tissues were grinded using the MagNA Lyser rotor (Roche Life Science) for 1–2 cycles depending on the size of the tissue, alternating between the MagNA Lyser rotor and wet ice. Tissue homogenates were briefly spun down to pellet the beads and supernatant was pipetted out for nucleic acid extraction. Supernatant was mixed with 1/10 bromochloropropane (BCP) to Tri-reagent volume. Mixture was vortexed and incubated for 5 min in room temperature, before spinning at 12,000 × *g* for 15 min at 4 °C to achieve phase separation. For RNA extraction, the upper aqueous layer was pipetted into a new tube containing 12 μL of glycogen (Thermo Scientific) and 0.5 mL isopropanol. Sample was inverted to mix and spun at 15,000 × *g* for 10 min in room temperature. Isopropanol was carefully discarded without losing pellet, and then washed with 0.7 mL 75% ethanol twice by spinning at 15,000 × *g* for 10 min at room temperature. After discarding the ethanol, RNA pellet was dried in room temperature for 10–15 min. Finally, RNA resuspension buffer (10 mM Tris, pH 8) was added to the RNA-containing tube and incubated in 37 °C heat block for 15 min to elute. Resuspended RNA was stored in −80 °C until assayed. For DNA extraction, the interphase and organic layers, not used for RNA extraction, were mixed with 0.5 mL of DNA extraction buffer, containing 4 M guanidine thiocyanate (Sigma), 50 mM sodium citrate (Sigma), and 1 M Tris base. Reaction was vortexed and incubated for 5 min in room temperature, before spinning at 12,000 × *g* for 15 min 4 °C to achieve phase separation. DNA-containing upper aqueous layer was pipetted into a new tube containing 12 μL of glycogen (Thermo Scientific) and 0.4 mL isopropanol. The remaining steps follow RNA extraction protocol described above, with the exception of the DNA resuspension buffer (10 mM Tris, pH 9) used to elute the DNA pellet.

Viral copies were measured by quantitative reverse transcription PCR (RT-qPCR) or qPCR that targets a highly conserved sequence of Gag^39^. The assays used the SGAG21 forward primer (GTCTGCGTCATPTGGTGCATTC), SGAG22 reverse primer (CACTAGKTGTCTCTGCACTATPTGTTTTG), and pSGAG23 probe (5′-6-carboxyfluorescein [FAM]-CTTCPTCAGTKTGTTTCACTTTCTCTTCTGCG-black hole quencher [BHQ1]-3′) (Supplementary Table 4). All viral detection assays were performed by members of the ONPRC Molecular Virology Core, who were blinded in the treatment conditions of each animal.

To quantitate SHIV viral RNA in plasma, RT-qPCR reactions were performed using the TaqMan Fast Virus 1-Step Master Mix (Applied Biosystems). The reactions used the total RNA extracted from 300 μL of plasma, with 900 nM SGAG21, 900 nM of SGAG22, and 250 nM pSGAG23 in a final volume of 30 μL. Viral RNA copies per reaction were calculated with a standard curve created by using in vitro transcribed SIVgag RNA that was serially diluted in 5 ng/μL yeast tRNA (Sigma R5636). SIV-positive plasma RNA was used as positive control and nuclease free water was used as negative control. Reactions were run with the Applied Biosystems QuantStudio 6 Flex instrument (Life Technologies) using the following thermal conditions: 50 °C for 5 min; 95 °C for 20 s; [95 °C for 3 s, 60 °C for 30 s] × 45 cycles. The limit of quantification for this assay is 50 copies/mL.

To detect SHIV viral RNA copies in cell pellets and tissues, a two-step RT-qPCR reaction was performed^40^, where 2.5 μg RNA was synthesized to complementary DNA (cDNA) using 20 U Superscript II RT (Thermo Fisher/Invitrogen), 5 mM MgCl_2_, 0.5 mM dNTPs, 1 mM dithithreitol (DTT), 150 ng random hexamers, 1× TaqMan PCR buffer (with 0.05% gelatin and 0.02% Tween-20), and 20 U RNAaseOut in a final volume of 30 μL. The reaction was performed with an Applied Biosystems ABI 9700 instrument using the following thermal conditions: 25 °C for 15 min, 42 °C for 40 min, 90 °C for 15 min, 25 °C for 30 min, and 5 °C hold. Following reverse transcription, qPCR was performed by adjusting the reaction to contain 1.25 U Platinum Taq (Applied Biosystems), 600 nM of SGAG21, 600 nM of SGAG22, 100 nM pSGAG23, 1× TaqMan PCR II buffer, 4.5 mM MgCl_2_, and 50 nM ROX passive reference dye, in a final volume of 50 μL. Viral RNA copies per reaction were calculated with a standard curve created by using in vitro transcribed SIVgag RNA that was serially diluted in 100 ng/μL yeast tRNA (Sigma R5636). Reactions were performed in an Applied Biosystems ABI 7500 instrument using the following thermal condition: 95 °C for 2 min; [95 °C for 15 s, 60 °C for 1 min] × 45 cycles. To detect SHIV viral DNA copies in cell pellets and tissues, qPCR reactions were done using Taqman Fast Advanced Master Mix (Life Technologies). Extracted DNA was first heated at 95 °C for 5 min and then placed on ice. The reactions used 2.5 μg of DNA, with 600 nM of SGAG21, 600 nM of SGAG22, and 100 nM pSGAG23. Viral DNA copies per reaction were calculated with a standard curve created with linearized plasmid DNA containing the SIVgag sequence that was serially diluted in TE buffer containing 2.5 ng/mL SIV-negative rhesus genomic DNA as carrier. Reactions were run with the Applied Biosystems QuantStudio 6 Flex instrument (Life Technologies) using the following thermal conditions: 50 °C for 2 min; 95 °C for 20 s; [95 °C for 1 s, 60 °C for 20 s] × 45 cycles. The limit of quantification for this assay is 10 copies/million cells for cell pellets and 7 copies/million cells for whole tissue biopsies.

### Leronlimab measurement in plasma and tissues

Enzyme-linked immunosorbent assay (ELISA) was used to detect free Leronlimab in plasma. Half-area 96-well Costar Assay Plates (Corning) were coated with the anti-idiotype antibody PA-22, provided by CytoDyn, at 1.5 μg/mL in carbonate-bicarbonate buffer (Thermo Fisher) and incubated overnight. Plates were washed three times with PBS-T (PBS + 0.1% Tween-20) and blocked with blocking buffer (PBS + 0.4% Tween-20 + 10% BSA) for at least 2 h at RT. Leronlimab concentration was calculated with a standard curve created with a serial titration of Leronlimab diluted in blocking buffer with a range of 4.7–300 ng/mL. Heat-inactivated plasma samples were also diluted with blocking buffer. After incubating for 30 min at RT, plates were washed three times with 0.5 M NaCl in PBS and incubated with 1:20,000 dilution of mouse anti-human IgG_4_ pFc′-horseradish peroxidase (HRP) (Southern Biotech) in blocking buffer for 30 min at RT. Plate was washed three times with PBS-T and developed for 2 min using 3,3′,5,5′-tetramethylbenzidine (TMB) substrate (Southern Biotech). Reaction was stopped with 1 N H_2_SO_4,_ Plates were read on the Synergy HTX Milti-Mode Microplate Reader (BioTek) and data were collected using software Gen5 v3.09 at two absorbance wavelengths: 650 nm for the developing reaction and 450 nm for the developed reaction after the reaction was stopped with 1 N H_2_SO_4_. Final OD was determined by OD_450 nm_ minus OD_650 nm_. The limit of detection for the assay is 22.5 ng/mL. Leronlimab in tissue was quantified in supernatants prepared from tissue homogenates by ELISA as described above. Further, the mass of the total protein in the collected tissues was determined by the Pierce Coomassie Plus Broadford Assay (Thermo Fisher) following the manufacturer’s instructions. Tissue concentration of Leronlimab is reported as the ng of Leronlimab per mg of total protein.

### Measurement of Leronlimab antidrug antibodies (ADA)

Half-area 96-well Costar Assay Plates (Corning) were coated with 2 μg/mL Leronlimab (Cytodyn, Vancouver, WA). Plates were washed with PBS-T three times and blocked with blocking buffer for 2 h at RT. Plates were then washed three times with PBS-T. Heat-inactivated plasma samples were serially diluted in blocking buffer, added to the plates in duplicate, and incubated at RT for 30 min. Plates were then washed three times with 0.5 M NaCl in PBS. To determine ADAs from RM, a secondary antibody recognizing rhesus IgG (anti-rhesus IgG1/3[1B3]-HRP, NHP Reagent Resource) and conjugated to HRP was added. Plates were incubated at RT for 30 min, and then washed three times with PBS-T. TMB solution (Southern Biotech) was added at RT for 2 min and the reaction was stopped with 1 N H_2_SO_4_. Absorbance was read at 450 nm on a Synergy plate reader (BioTek). ADA titers are defined as the reciprocal of the highest dilution of the sample that yields a positive result (e.g. dilution of 1/2460 = titer of 2460). A positive result was defined as twice that of background values.

### Env sequencing

Viral sequencing and analysis were adapted from previously published genome-wide SIVmac239 sequencing protocols. Viral RNA was isolated from virus stocks and plasma samples using QIAamp MinElute Virus Spin Kit following the manufacturer’s instructions. Complementary DNA was generated with the SuperScript III One-Step RT-PCR with Platinum Taq (Thermo Fisher). SHIV *env* forward primer (GGCATAGCCTCATAAAATATCTG) and the SHIV *env* reverse primer (ACAGAGCGAAATGCAGTGATATT) were used to amplify a ~4.5 kb amplicon spanning the *env* gene (Supplementary Table 4). RT-PCR reactions were performed on Eppendorf Mastercycler Pro S Thermal Cyclers using the following thermal conditions: 50 °C for 30 min; 94 °C for 2 min; [94 °C for 15 s, 60 °C for 1 min, 68 °C for 4 min] × 2 cycles; [94 °C for 15 s, 58 °C for 1 min, 68 °C for 4 min] × 2 cycles; [94 °C for 15 s, 60 °C for 1 min, 68 °C for 4 min] × 45 cycles; 68 °C for 10 min; and hold at 4 °C. The resulting 4.9 kb fragments were purified on a 1% agarose gel and purified using NucleoSpin Gel and PCR Clean-up Kit (Macherey-Nagel). Dual-indexed Illumina MiSeq-compatible libraries were then prepared using the Nextera XT DNA Sample Prep Kit, and purified with AMPure XP magnetic beads (Beckman Coulter). Libraries were analyzed on an Agilent 2100 Bioanalyzer using the HS DNA kit (Agilent), normalized to 2 nM, pooled at an equimolar ratio, and sequenced in parallel on the Illumina MiSeq. Sequence reads were processed^41,42^, where raw data were trimmed using Trimmomatic version 0.39 and aligned to the SHIVSF_162_ reference sequence (GenBank Accession No. KF042063.1) using BWA-MEM version 0.7.17-r1188 (refs. ^43,44^). All bases of the alignment were evaluated and single-nucleotide polymorphisms (SNPs) and deletion/insertion polymorphisms were called for bases with a quality score above 17. Importantly, the identity of the associated read was retained for each SNP, which allowed the phase of SNPs to be considered. This information allowed amino acid translations to be calculated based on the sequence of each individual read, as opposed to the consensus sequence. SNP analysis and visualization of mutations was performed using the SequenceAnalysis module, written for LabKey Server 21.3 (ref. ^45^).

### T cell assays

SHIV-specific CD8+ T cell responses in PBMC were measured by flow cytometric intracellular cytokine staining (ICS)^4^. 1 × 10^6^ PBMCs were incubated with overlapping 15-mer peptide pools spanning SIVmac239 Gag or Vif open reading frame and co-stimulated with CD28 and CD49d antibodies (eBiosciences) for 1 h, followed by the incubation with Brefeldin A (Sigma-Aldrich) for an additional 8 h. Stimulation with rhesus cytomegalovirus (RhCMV) lysate served as a positive control while incubation without antigen served as background control. Cells were surface stained with antibodies for CD3, CD4, CD8, and amine-reactive dye, fixed with 2% PFA, permeabilized with BD FACS Lysing Solution (BD Biosciences), and stained intracellularly for IFN-γ, TNF-α, and CD69. Samples were collected on LSR-II instrument and FACsDIVA version 6.1 and analyzed with FlowJo v10 (Tree Star) by gating on singlet, live, CD3+, CD4−, and CD8+ cells. Responding CD8+ T cells were measured by Boolean gating on cells that are CD69+/TFN-α+ and/or CD69+/IFN-γ+.

### Adoptive transfer

To confirm sterilizing protection, cells from the infected control animals (all six animals) or uninfected Leronlimab-treated animals (four animals in the 10 mg/kg group and all six animals in the 50 mg/kg group) were adoptively transferred into one SHIV-naïve RM per animal group, as detailed in Table S5. Cells were prepared an hour before infusion by resuspending in 1 mL of Hank’s Buffered Salt Solution (HBSS) with 15 U/mL heparin. Recipient RM were sedated with ketamine HCl (8–20 mg/kg) or Telazol (2–5 mg/kg) and prophylactically treated with Benadryl (5 mg/kg) prior to infusion of donor cells. Donor cells were slowly infused intravenously with an infusion pump at a maximum rate of 22 mL/kg/h. Animals were monitored for at least 2 h for post-procedural complications.

### Antibodies

The following conjugated antibodies were used in these studies: (a) from BD Biosciences, D058-1283 (CD45; PE Cy7; 1:100; cat# 561294), SP34-2 (CD3; Alexa 700; 1:100; cat# 557917), SP34-2 (CD3; PE; 1:20; cat# 552127), LP200 (CD4; PerCP-Cy5.5; 1:40; cat# 552838), RPA-T8 (CD8; PacBlu; 1:40; cat# 558207), SK1 (CD8; TruRed; 1:100; cat# 341051), 3GB (CD16; Alexa 700; 1:100; cat# 560713), 25723.11 (IFN- γ; APC; 1:100; cat# 502512), 6.7 (TNF-α; PE; 1:100; cat# 554513), 3A9 (CCR5; APC; 1:100; cat# 560748), SK1 (CD8; BUV737; 1:50; cat# 612754), L200 (CD4; BUV395; 1:200; cat# 564107), FN50 (CD69; PE-Texas Red; 1:100; cat# 562617), SP34-2 (CD3; Pacific Blue; 1:100; cat# 558124); (b) from BioLegend, OKT4 (CD4; APC-Cy7; 1:100; cat# 305612), RPA-T4 (CD4; APC; 1:100; cat# 300537); (c) from Beckman Coulter, RMO52 (CD14; PE-Texas Red; 1:40; cat# IM2707U), KC57 (HIV Gag p24; FITC; 1:100; cat# 6604665); (d) from Sigma, HP-6025 (IgG4; FITC; 3:100; cat# F9890); and (e) from SouthernBiotech, HP6023 (mouse anti-human IgG_4_ pFc′; HRP; 1:20,000; cat# 9190-05); (f) from NHP Reagent Resource, 1B3 (anti-rhesus IgG1/3; HRP; 1:5000). The following unconjugated antibodies were used: (a) 55-2F12 (SIV Gag p27; NIH AIDS Research and Reference Reagent Program), conjugated in-house to FITC using Pierce^TM^ FITC Antibody Labeling Kit (Thermo Fisher) and used at approximately 1:100 depending on the efficacy of conjugation; (b) PA-14 (Leronlimab; CytoDyn), conjugated in-house to PacBlu using Pacific Blue^TM^ Antibody labeling Kit (Thermo Fisher) and used at approximately 1:80 depending on the efficacy of conjugation; (c) anti-idiotype antibody, PA-22 (CytoDyn). Live/dead Fixable Yellow Dead Cell Stain Kit and Near-IR Dead Cell Stain Kit (Thermo Fisher) were amine-reactive dyes used at 1:1000 dilution to assess cell viability.

### Statistical analyses

Time to infection was assessed by log-rank test. Differences in CCR5 expression percentages were measured by nonparametric Kruskal–Wallis test, and differences in the number of CCR5 surface molecules were assessed by nonparametric Mann–Whitney test. Statistical significance was determined at the significant alpha level of 0.05. Statistical analyses were conducted using GraphPad Prism software version 6.0 (GraphPad Software, La Jolla, California).

### Reporting summary

Further information on research design is available in the Nature Research Reporting Summary linked to this article.

## Supplementary information

Supplementary Information

Reporting Summary

## Data Availability

SHIV_SF162P3_ sequence data that support the findings of this study have been deposited in GenBank with the accession code KF042063.1. All other relevant data that support the findings of this study are available from the corresponding authors upon reasonable request. Source data are provided with this paper.

## Source data

Source Data

## Code availability

Leronlimab is available via MTA from CytoDyn.
