# Supplementary material for: Antibody-based CCR5 blockade protects Macaques from mucosal SHIV transmission
Source: Nat Commun. 2021 Jun 7;12:3343. doi: 10.1038/s41467-021-23697-6 (PMC8184841; doi:10.1038/s41467-021-23697-6)
Supplement: Supplementary file 1 — Supplementary Information [file 41467_2021_23697_MOESM1_ESM.pdf]

Antibody-based CCR5 Blockade Protects Macaques from Mucosal SHIV Transmission

Chang et al.

SUPPLEMENTARY LIST:

Supplementary Figure 1. Leronlimab blocks spreading infection of CCR5-utilizing HIV *in vitro*.

Supplementary Figure 2. Leronlimab binds to CCR5 on human and macaque cells.

Supplementary Figure 3. Longitudinal complete blood count and serum chemistry.

Supplementary Figure 4. Longitudinal lymphocyte counts.

Supplementary Figure 5. Calculation of CCR5 receptor occupancy (RO).

Supplementary Figure 6. Plasma viremia comparison of SHIV-infected macaques.

Supplementary Figure 7. Assessment of V3 loop sequences from infected macaques.

Supplementary Figure 8. Longitudinal RhCMV lysate-specific CD8+ T cells responses.

Supplementary Figure 9. Flow cytometry gating strategy.

Supplementary Table S1. HIV-1 isolates utilized for *in vitro* spreading assay.

Supplementary Table S2. Demography of macaques.

Supplementary Table S3. Source of cells used in adoptive transfer.

Supplementary Table S4. Primer names and sequences.

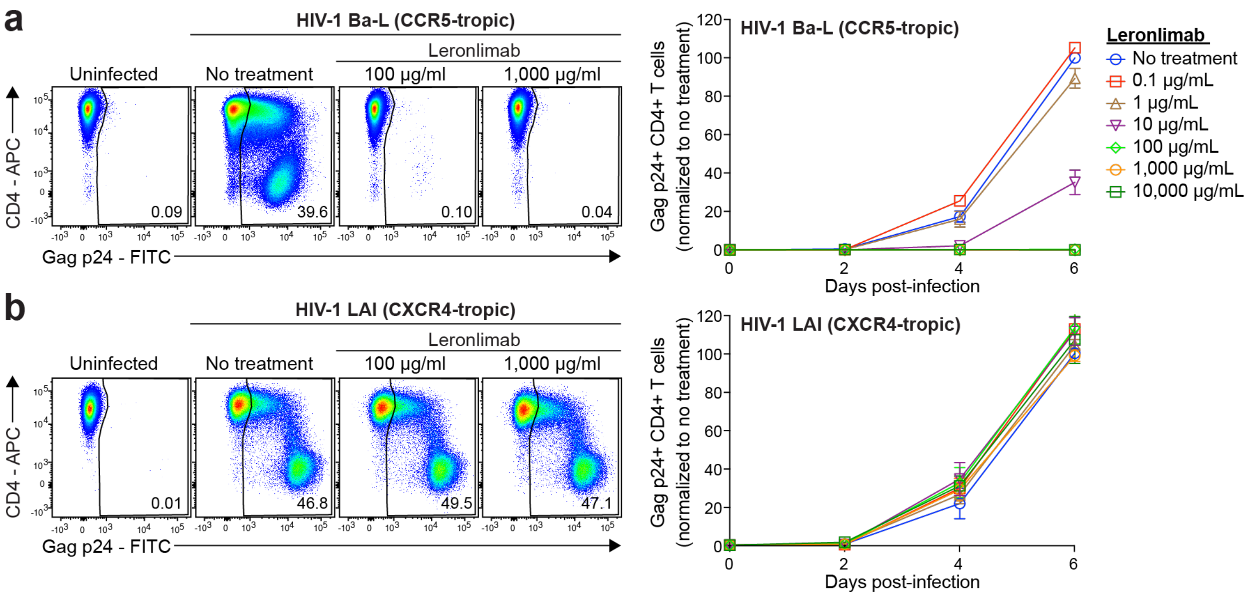

Supplementary Figure 1. Leronlimab blocks spreading infection of CCR5-utilizing HIV *in vitro*. CCR5 WT CD4+ T cells (n=3 donors) were infected and treated with or without Leronlimab at different concentrations. Cells were cultured for six days, and media (with Leronlimab at the desired concentration) was replenished on day 2 and day 4. On day 6, cells were stained for intracellular Gag protein. (Left) Representative flow cytometry plots displaying day 6 of the intracellular Gag p24 staining of **(a)** HIV-1 Ba-L, a CCR5-utilizing virus, **(b)** HIV-1 LAI, a CXCR4-utilizing virus, (Right) Summary (mean ±SEM) of the longitudinal infection, with all values normalized to day 6 “no treatment”. Source data are provided as a Source Data file.

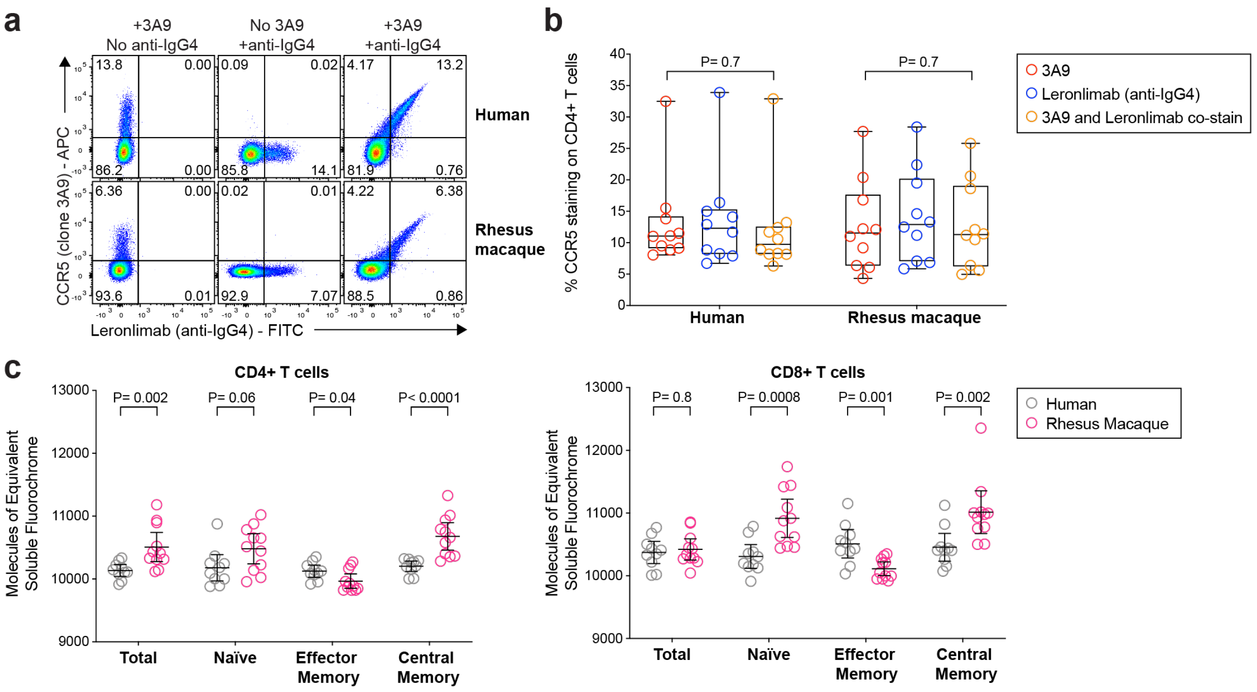

Supplementary Figure 2. Leronlimab binds to CCR5 on human and macaque cells. (a) Representative flow cytometry plots of CCR5 receptor staining on CD4+ T cells. Cells were stained with anti-CCR5 antibody (clone 3A9) only, Leronlimab only, or both antibodies. The secondary antibody, anti-human IgG4 (clone HP-6025) was used to stain Leronlimab. **(b)** Summary of the %CCR5 receptor on CD4+ T cells from human (n=10 donors) and macaque (n=10 donors) PBMC as determined by the three different staining methods. Box plots are presented where the middle line is the median, the lower and upper hinges correspond to 25th and 75th percentiles – interquartile range – and the upper and lower whiskers extend to the maximum and minimum values. Differences between the three staining methods was assessed by one-sided nonparametric Kruskal-Wallis test followed by Dunn's correction for multiple comparisons. **(c)** Summary (mean ±95% confidence interval) of the number of CCR5 receptors on total and memory subset CD4+ T cells (left) and CD8+ T cells (right) from human (n=10 donors) and macaque (n=10 donors) PBMC. Described in the Methods, the number of CCR5 receptors was equivalent to the number of detectable fluorochromes, using PE-conjugated Leronlimab. P-values calculated from two-sided nonparametric Mann-Whitney test. P-values <0.05 is significant. Source data are provided as a Source Data file.

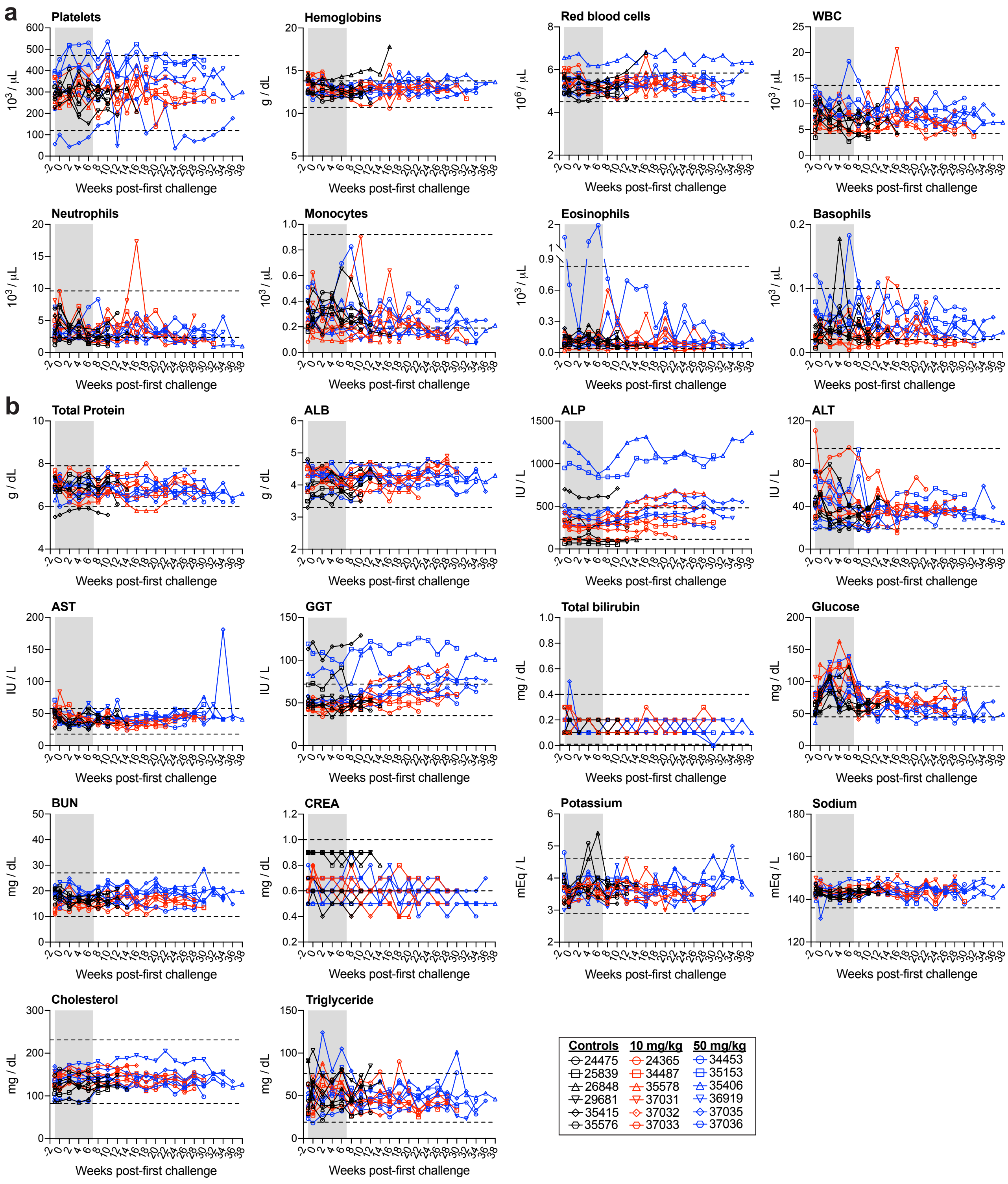

Supplementary Figure 3. Longitudinal compete blood count and serum chemistry. Longitudinal values are shown for control group (n=6, in black), 10 mg/kg group (n=6, in red), and 50 mg/kg group (n=6, in blue). The gray box indicates Leronlimab treatment period that started on study week -1 and ended on study week 7. Two dotted lines indicate the standard reference range for each parameter in rhesus macaques housed at ONPRC. **(a)** Peripheral

blood levels of platelets, hemoglobin, red blood cells, white blood cells (WBC), neutrophils, monocytes, eosinophils, and basophils. **(b)** Serum levels of total protein, albumin (ALB), alkaline phosphatase (ALP), alanine aminotransferase (ALT), aspartate aminotransferase (AST), gamma-glutamyltransferase (GGT), total bilirubin, glucose, blood urea nitrogen (BUN), creatinine (CREA), potassium, sodium, cholesterol, and triglyceride. Group colors and individual animal symbols are consistent throughout the manuscript. Source data are provided as a Source Data file.

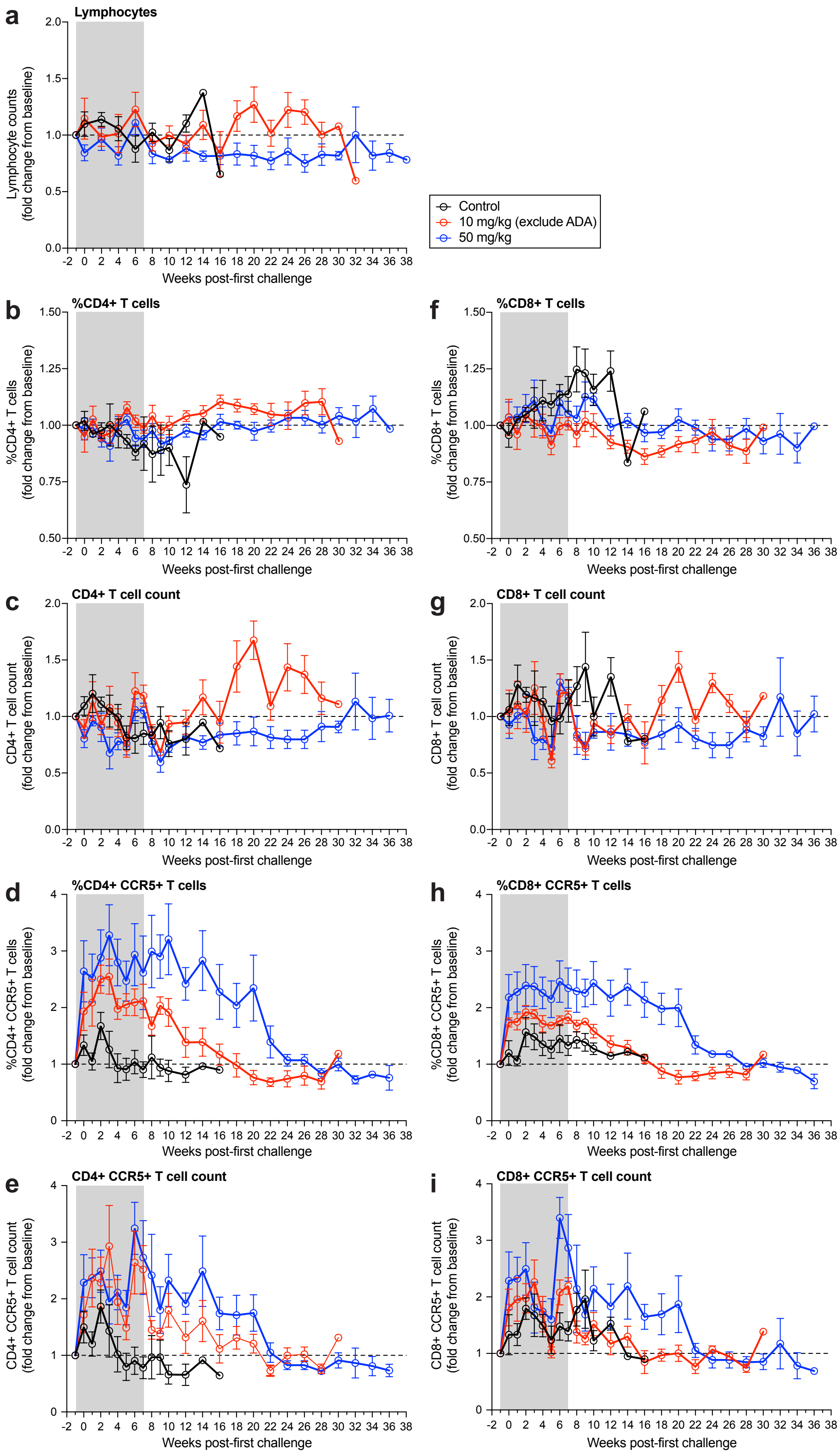
a
Lymphocytes
Lymphocyte counts (fold change from baseline)
Weeks post-first challenge
Control
10 mg/kg (exclude ADA)
50 mg/kg
b
%CD4+ T cells
%CD4+ T cells (fold change from baseline)
f
%CD8+ T cells
%CD8+ T cells (fold change from baseline)
c
CD4+ T cell count
CD4+ T cell count (fold change from baseline)
g
CD8+ T cell count
CD8+ T cell count (fold change from baseline)
d
%CD4+ CCR5+ T cells
%CD4+ CCR5+ T cells (fold change from baseline)
h
%CD8+ CCR5+ T cells
%CD8+ CCR5+ T cells (fold change from baseline)
e
CD4+ CCR5+ T cell count
CD4+ CCR5+ T cell count (fold change from baseline)
i
CD8+ CCR5+ T cell count
CD8+ CCR5+ T cell count (fold change from baseline)

Supplementary Figure 4. Longitudinal lymphocyte counts. Mean (± SEM) of control group (n=6, in black), 10 mg/kg group (n=5, excluding 37032, who developed ADA, in red), and 50 mg/kg group (n=6, in blue). The gray box indicates Leronlimab treatment period that started on study week -1 and ended on study week 7. Panels A-I show longitudinal peripheral blood values normalized to baseline value at study week -1. Dotted line at Y=1 denotes fold change from baseline for: **(a)** Lymphocyte counts. **(b)** Percentage of CD4+ T cells. **(c)** CD4+ T cell count. **(d)** Percentage of CD4+ CCR5+ T cells. **(e)** CD4+ CCR5+ T cell count. **(f)** Percentage of CD8+ T cells. **(g)** CD8+ T cell count. **(h)** Percentage of CD8+ CCR5+ T cells. **(i)** CD8+ CCR5+ T cell count. Study week -1 to week 7 was the period of Leronlimab treatment. Group colors are consistent throughout the manuscript. Source data are provided as a Source Data file.

a

$$\%RO = \frac{\%\ IgG4}{\%\ IgG4 + \%\ Leronlimab\text{-}PB} \times 100\%$$

b

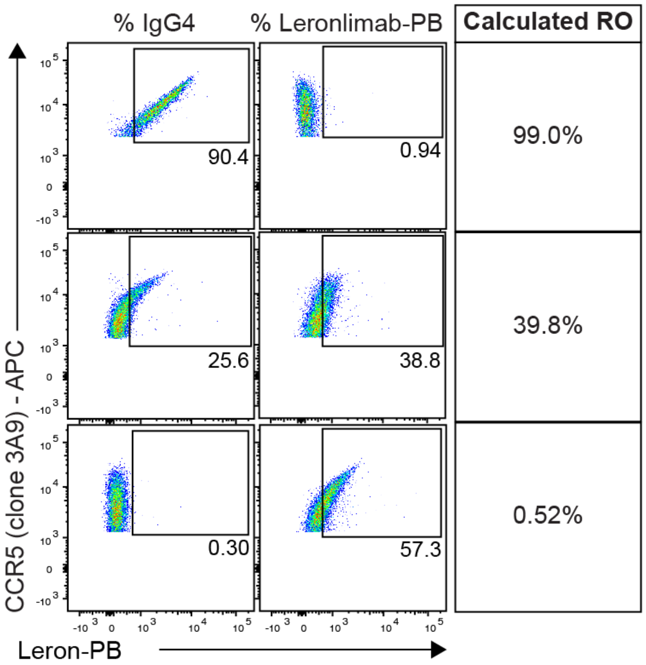

Supplementary Figure 5. Calculation of CCR5 receptor occupancy (RO). (a) Equation used to calculate CCR5 RO. **(b)** Representative flow cytometry plots displaying the different components required to calculate RO and different occupancy levels: fully occupied, partially occupied, and not occupied by Leronlimab. Briefly, cells were gated on CD45+, singlet, live, CD3+, CD4+/CD8-, CCR5+, and either IgG4+ or Leronlimab-PB+ events. Table at right shows the calculated percentage of CCR5 RO.

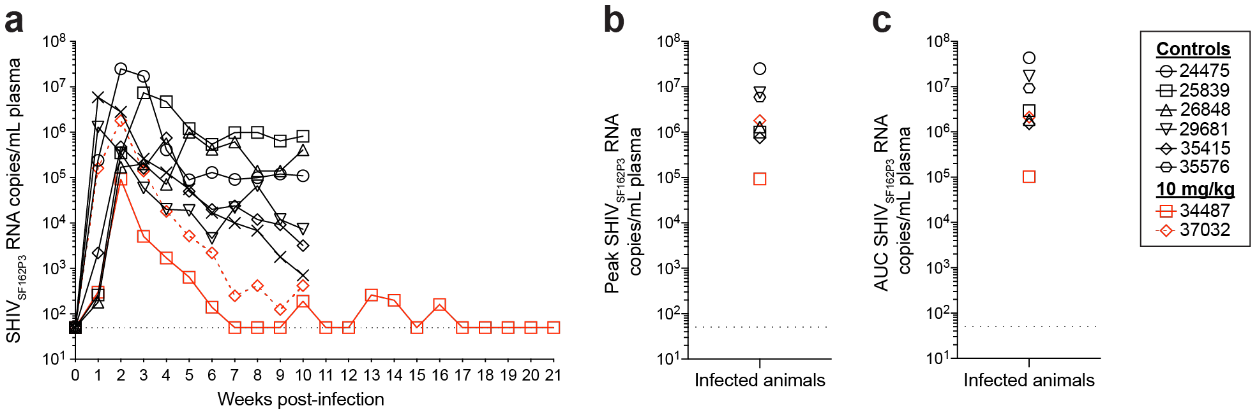

Supplementary Figure 6. Plasma viremia comparison of SHIV-infected macaques. Legend at right shows symbols used in panels A-C identifying individual rhesus macaques. Horizontal dashed line denotes assay limit of quantification (50 copies/ml); undetectable plasma viral loads graphed as 50 copies/mL**. (a)** Longitudinal SHIV$_{SF162P3}$ plasma viral loads shown in weeks post-infection. Six infected control animals and one 10 mg/kg animal (37032) that developed ADA were euthanized at ten weeks after infection while one 10 mg/kg animal (34487) was euthanized after five consecutive weeks of aviremia. **(b)** Summary of highest SHIV$_{SF162P3}$ plasma viral load for each infected animal. **(c)** Summary of area under the curve (AUC) for each infected animal. Group colors and individual animal symbols are consistent throughout the manuscript. Source data are provided as a Source Data file.

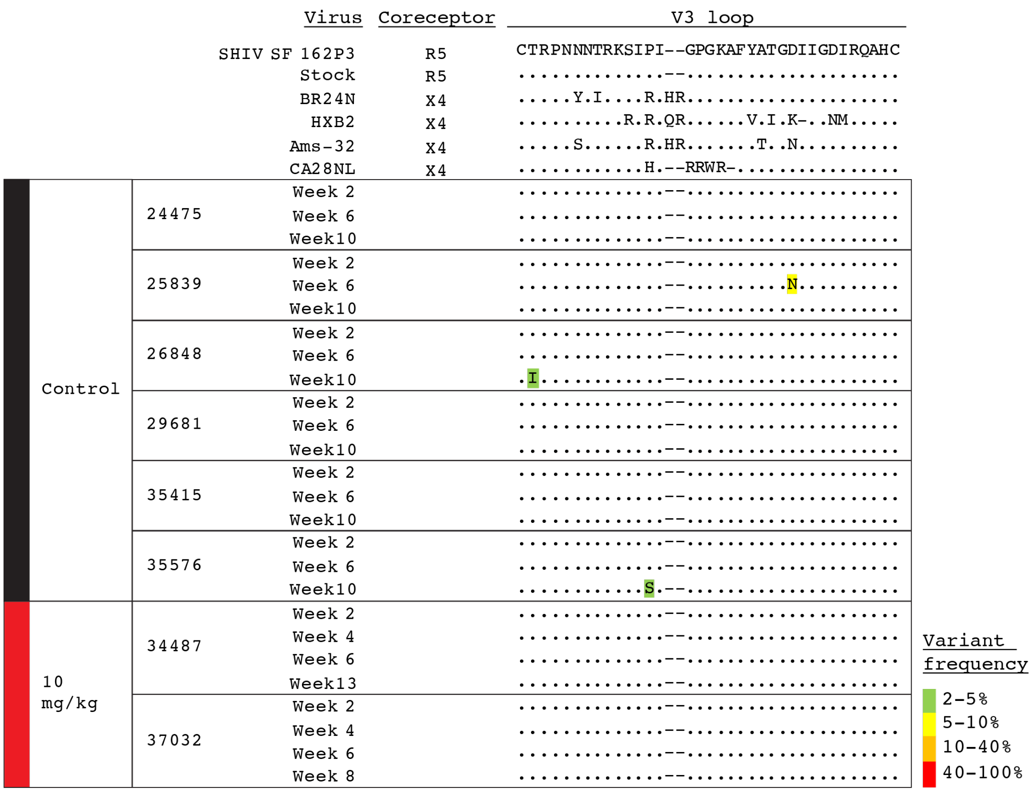

Supplementary Figure 7. Assessment of V3 loop sequences from infected macaques in PrEP study. The *env* gene was sequenced from plasma RNA at three timepoints post-infection: week 2, 6, and 10. “SHIV SF 162P3” is the theoretical consensus sequence. “Stock” is the sequence of the stock virus used for viral challenges. “BR24N”, “HXB2”, “Ams.32”, and “CA28NL” are sequences of CXCR4-ultilizing isolates related closely to $SHIV_{SF162P3}$. All sequences are aligned to “SHIV SF 162P3”, where the dots denote similarities and the dashes indicate gaps. Legend at right denotes the variant frequency. Variants <2% were excluded. Group colors are consistent throughout the manuscript. Source data are provided as a Source Data file.

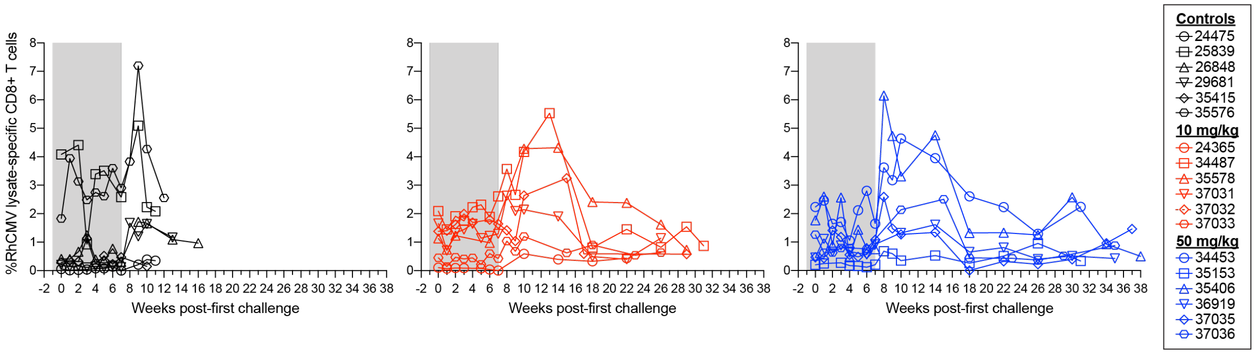

Supplementary Figure 8. Longitudinal RhCMV lysate-specific CD8+ T cells responses. In the same ICS experiment to determine SHIV-specific CD8+ T cell responses (Figure 2c), longitudinal PBMC samples were stimulated with RhCMV lysate as a positive control. Cells were stained and collected on a flow cytometer as described in the Methods. Using FlowJo v10, cells were gated for live, CD3+, CD4-, and CD8+. Percentage of RhCMV lysate-specific responses were determined by Boolean gating to cells that were CD69+/TFN-α+ and/or CD69+/IFN-γ+. Summary of longitudinal changes of RhCMV lysate-specific CD8+ T cell responses for the Control animals (left), 10 mg/kg treated animals (middle), and 50 mg/kg treated animals (right). Gray box denotes the Leronlimab treatment phase for Leronlimab-treated macaques. Group colors and individual animal symbols are consistent throughout the manuscript. Source data are provided as a Source Data file.

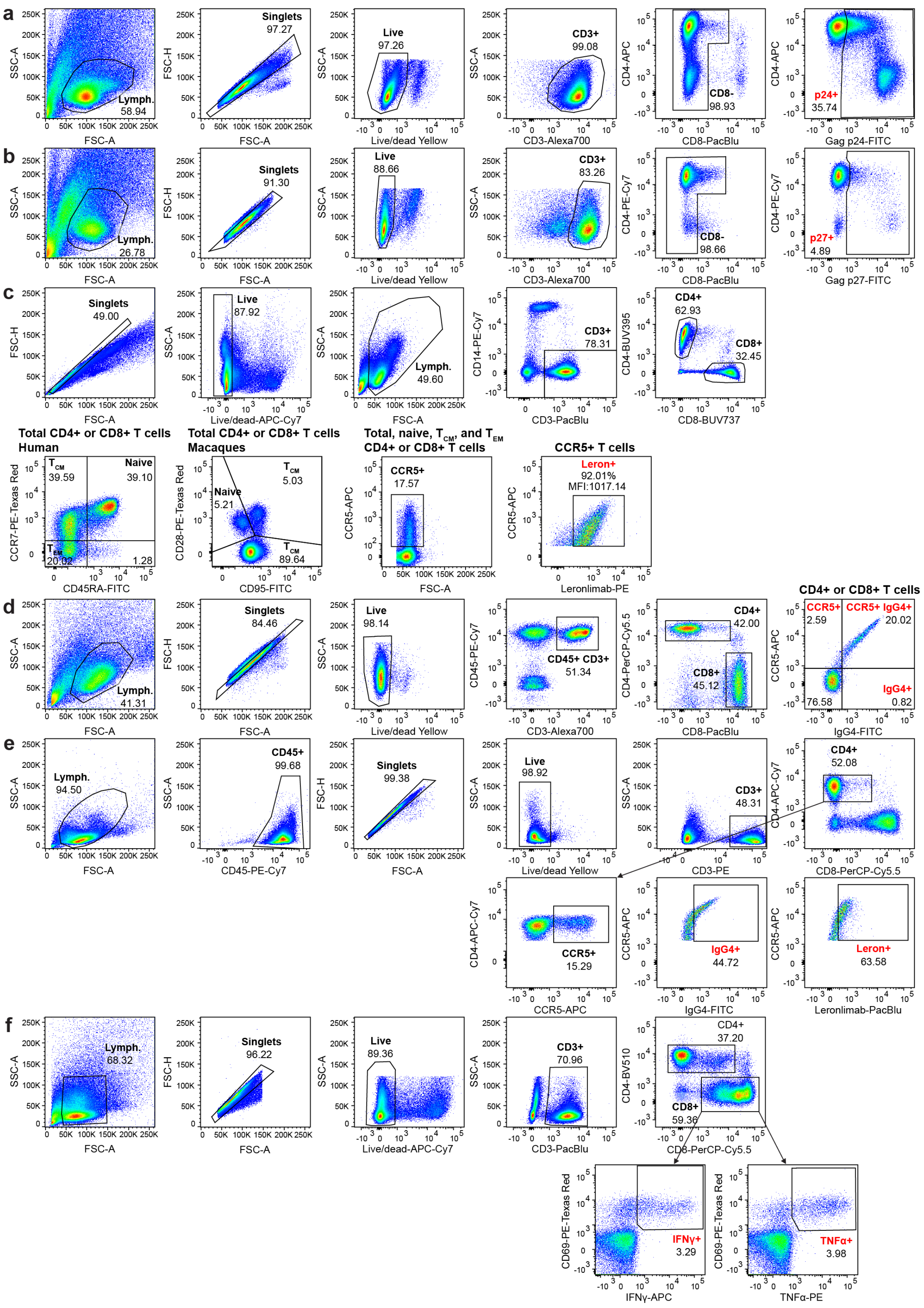

a
Lymph.
58.94
Singlets
97.27
Live
97.26
CD3+
99.08
CD8-
98.93
p24+
35.74
SSC-A
FSC-A
FSC-H
Live/dead Yellow
CD3-Alexa700
CD4-APC
CD8-PacBlu
Gag p24-FITC
b
Lymph.
26.78
Singlets
91.30
Live
88.66
CD3+
83.26
CD8-
98.66
p27+
4.89
CD4-PE-Cy7
Gag p27-FITC
c
Singlets
49.00
Live
87.92
Lymph.
49.60
CD3+
78.31
CD4+
62.93
CD8+
32.45
Live/dead-APC-Cy7
CD14-PE-Cy7
CD3-PacBlu
CD4-BUV395
CD8-BUV737
Total CD4+ or CD8+ T cells
Human
Total CD4+ or CD8+ T cells
Macaques
Total, naive, TCM, and TEM
CD4+ or CD8+ T cells
CCR5+ T cells
TCM
39.59
Naive
39.10
TEM
20.02
1.28
CCR7-PE-Texas Red
CD45RA-FITC
TCM
5.03
Naive
5.21
TCM
89.64
CD28-PE-Texas Red
CD95-FITC
CCR5+
17.57
CCR5-APC
Leron+
92.01%
MFI:1017.14
Leronlimab-PE
d
Lymph.
41.31
Singlets
84.46
Live
98.14
CD45+ CD3+
51.34
CD4+
42.00
CD8+
45.12
CD4+ or CD8+ T cells
CCR5+
2.59
CCR5+ IgG4+
20.02
IgG4+
0.82
76.58
CD45-PE-Cy7
CD4-PerCP-Cy5.5
IgG4-FITC
e
Lymph.
94.50
CD45+
99.68
Singlets
99.38
Live
98.92
CD3+
48.31
CD4+
52.08
CD45-PE-Cy7
CD3-PE
CD4-APC-Cy7
CD8-PerCP-Cy5.5
CCR5+
15.29
IgG4+
44.72
Leron+
63.58
CCR5-APC
Leronlimab-PacBlu
f
Lymph.
68.32
Singlets
96.22
Live
89.36
CD3+
70.96
CD4+
37.20
CD8+
59.36
CD4-BV510
IFNγ+
3.29
TNFα+
3.98
CD69-PE-Texas Red
IFNγ-APC
TNFα-PE

Supplementary Figure 9. Flow cytometry gating strategy. Representative flow cytometry gating strategy to determine the a) percentage of intracellular Gag p24 presented on Fig. 1a-b and Supplementary Fig. 1a-b; b) percentage of intracellular Gag p27 presented on Fig. 1c; c) number of CCR5 receptors presented on Supplementary Fig. 2c; d) percentages for CCR5, Leronlimab (by the secondary antibody, anti-human IgG4-FITC), CD4+ T cells, and CD8+ T cells presented on Fig. 3a, Supplementary Fig. 2a-b, and Supplementary Fig. 4a-i; e) percentage of CCR5 receptor occupancy used in Fig. 2c, Fig. 3a, and Supplementary Fig. 5b; f) percentage of ICS used in Fig. 4c and Supplementary Fig. 8.

Co-receptor	HIV-1	Subtype/CRF	GenBank No.	TCID50 used per infection
CCR5	HIV-1 KNH1088	A	AF457063	2.80E+01
	HIV-1 KSM4030	A	AF457079	3.15E+01
	HIV-1 91/US/1 (GS004)	B	AY173952	6.49E+03
	HIV-1 Ba-L	B	AY713409	8.24E+04
	HIV-1 91/US/4	B	AY173955	4.09E+03
	HIV-1 93/MW/965	C	AY713413	1.42E+01
	HIV-1 20635-4	C	AY713414	7.56E+01
	HIV-1 90/SE/364	C	AY713416	1.30E+04
	HIV-1 57128	D	AF484502	1.01E+01
	HIV-1 A03349M1	D	AF484518	4.56E+02
	HIV-1 J32228M4	D	AF484516	5.56E+01
CXCR4	HIV-1 92UG029	A	AY713407	3.65E+03
	HIV-1 LAI	B	K03455	8.67E+03
	HIV-1 MN	B	M17449	2.39E+01
	HIV-1 89/BZ/167	B	AY173956	4.83E+02
	HIV-1 90/TH/BK132	B	AY173951	1.33E+02
	HIV-1 BK132/GS/009	B	AY173951	1.46E+04
	HIV-1 93/UG/065	D	AY713418	3.20E+00
	HIV-1 0503M02138	CRF01_AE	AY713424	6.67E+02
	HIV-1 NI1052	CRF01_AE	AY713423	7.60E+01
	HIV-1 NP1695	CRF01_AE	AY713419	1.33E+02
Dual	HIV-1 93RW024	A	AY713406	7.25E+03
	HIV-1 KER2008	A	AF457052	4.60E+03
	HIV-1 A018A	B	U12738	1.24E+05
	HIV-1 N119	B	HQ898266	4.50E+05
	HIV-1 NP1525	CRF01_AE	AY713420	9.05E+01

Supplementary Table 1. HIV-1 isolates utilized for *in vitro* spreading assay. Table showing the isolates used in the spreading assays with information on co-receptor tropism, subtype, GenBank, and $TCID_{50}$ used to infect targets in the assay shown in Figure 1.

Group	Animal	Gender	Age (Years)	MHC	
				B*08	B*17
Control	24475	F	15.60	-	-
	25839	M	13.52	-	-
	26848	M	12.54	-	-
	29681	M	9.44	-	-
	35415	M	3.26	+	-
	35576	F	3.58	-	-
10 mg/kg	24365	F	15.48	-	-
	34487	M	4.95	-	-
	35578	M	3.83	-	-
	37031	M	3.76	-	-
	37032	F	3.53	-	-
	37033	M	3.79	-	-
50 mg/kg	34453	F	4.65	-	-
	35153	M	3.81	-	-
	35406	M	3.80	-	-
	36919	M	3.08	-	-
	37035	M	2.96	-	-
	37036	F	3.00	-	-

Supplementary Table 2. Demography of macaques. Table showing the animal ID, gender, age, and MHC type for animals used in the PrEP study. Note the presence of one Mamu-B*08+ animal in the control group.

Animal group	Recipient animal ID	Donor animal ID	Axillary LN	Inguinal LN	Mesenteric LN	Bone Marrow	Spleen	Tissue distribution per animal	Numbers of adoptively transferred cells
Control	26047	24475	4%	4%	4%	0%	4%	**16%**	**7.8E+08**
		25839	4%	4%	4%	0%	4%	**16%**	
		26848	1%	4%	8%	0%	4%	**16%**	
		29681	4%	6%	3%	0%	4%	**17%**	
		35415	4%	4%	4%	0%	4%	**16%**	
		35576	4%	3%	4%	4%	4%	**19%**	
10 mg/kg	34279	24365	2%	1%	5%	0%	16%	**24%**	**6.9E+08**
		35578	5%	4%	10%	1%	5%	**26%**	
		37031	5%	3%	10%	1%	5%	**25%**	
		37033	5%	3%	10%	3%	5%	**26%**	
50 mg/kg	37037	34453	4%	4%	4%	4%	4%	**18%**	**9.7E+08**
		35153	4%	4%	4%	4%	4%	**18%**	
		35406	4%	4%	4%	1%	4%	**16%**	
		36919	2%	3%	3%	2%	4%	**15%**	
		37035	3%	3%	4%	4%	4%	**17%**	
		37036	1%	1%	4%	4%	7%	**17%**	

Supplementary Table 3. Source of cells used in adoptive transfer. Table showing the source of donor cells infused into each adoptive transfer recipients, with one recipient per experiment group. From the control group, all infected animals were donors. From the 10 mg/kg group, four aviremic animals were donors, excluding 34487 and 37032. From the 50 mg/kg group, all aviremic animals were donors. Mononuclear cells came from axillary lymph node, inguinal lymph node, mesenteric lymph node, bone marrow, or spleen. Shown are the percentage of combined tissues coming from each donor animal and the total cell count adoptively transferred to each recipient animal. Source data are provided as a Source Data file.

Primer Name	Primer Sequence (5’ – 3’)	Purpose
SGAG21 Forward	GTCTGCGTCATPTGGTGCATTC	Viral load quantitation in plasma, PBMC, tissues
SGAG22 Reverse	CACTAGKTGTCTCTGCACTATPTGTTTTG	Viral load quantitation in plasma, PBMC, tissues
pSGAG23 probe	5′-6-carboxyfluorescein [FAM]-CTTCPTCAGTKTGTTTCACTTTCTCTTCTGCG-black hole quencher [BHQ1]-3′	Viral load quantitation in plasma, PBMC, tissues
SHIV *env* Forward	GGCATAGCCTCATAAAATATCTG	Sequencing SHIV *env*
SHIV *env* Reverse	ACAGAGCGAAATGCAGTGATATT	Sequencing SHIV *env*

Supplementary Table 4. Primer names and sequences. List of primers used in this study.
