# Supplementary material for: Antibody-based CCR5 blockade protects Macaques from mucosal SHIV transmission
Source: Nat Commun. 2021 Jun 7;12:3343. doi: 10.1038/s41467-021-23697-6 (PMC8184841; doi:10.1038/s41467-021-23697-6)
Supplement: Supplementary file 2 — Reporting Summary [file 41467_2021_23697_MOESM2_ESM.pdf]

nature research

Corresponding author(s): Jonah Sacha and Lishomwa Ndhlovu

Last updated by author(s): April 30, 2021

Reporting Summary

Nature Research wishes to improve the reproducibility of the work that we publish. This form provides structure for consistency and transparency in reporting. For further information on Nature Research policies, see our Editorial Policies and the Editorial Policy Checklist.

Statistics

For all statistical analyses, confirm that the following items are present in the figure legend, table legend, main text, or Methods section.

n/a	Confirmed	
☐	☒	The exact sample size (n) for each experimental group/condition, given as a discrete number and unit of measurement
☐	☒	A statement on whether measurements were taken from distinct samples or whether the same sample was measured repeatedly
☐	☒	The statistical test(s) used AND whether they are one- or two-sided<br>*Only common tests should be described solely by name; describe more complex techniques in the Methods section.*
☒	☐	A description of all covariates tested
☒	☐	A description of any assumptions or corrections, such as tests of normality and adjustment for multiple comparisons
☐	☒	A full description of the statistical parameters including central tendency (e.g. means) or other basic estimates (e.g. regression coefficient) AND variation (e.g. standard deviation) or associated estimates of uncertainty (e.g. confidence intervals)
☐	☒	For null hypothesis testing, the test statistic (e.g. F, t, r) with confidence intervals, effect sizes, degrees of freedom and P value noted<br>*Give P values as exact values whenever suitable.*
☒	☐	For Bayesian analysis, information on the choice of priors and Markov chain Monte Carlo settings
☒	☐	For hierarchical and complex designs, identification of the appropriate level for tests and full reporting of outcomes
☒	☐	Estimates of effect sizes (e.g. Cohen's d, Pearson's r), indicating how they were calculated

Our web collection on statistics for biologists contains articles on many of the points above.

Software and code

Policy information about availability of computer code

Data collection	BD FACsDIVA version 6.1 (BD Biosciences, San Jose, CA, USA) for flow cytometry; Gen5 v3.09 for reading ELISA plate reading.
Data analysis	Flow Jo (Tree Star, Inc.), version 10<br>GraphPad Prism (GraphPad Software), version 6.0<br>BWA-MEM version 0.7.17-r1188<br>LabKey Server 21.3<br>Trimmomatic version 0.39

For manuscripts utilizing custom algorithms or software that are central to the research but not yet described in published literature, software must be made available to editors and reviewers. We strongly encourage code deposition in a community repository (e.g. GitHub). See the Nature Research guidelines for submitting code & software for further information.

Data

Policy information about availability of data

All manuscripts must include a data availability statement. This statement should provide the following information, where applicable:

- Accession codes, unique identifiers, or web links for publicly available datasets
- A list of figures that have associated raw data
- A description of any restrictions on data availability

The source data underlying Figs. 1-4, Supplementary Figs. S1-4, 6-8, and Supplementary Table 3 are provided as a Source Data file with this paper. SHIVSF162P3 sequence data that support the findings of this study has been deposited in GenBank with the accession code KF042063.1 (https://www.ncbi.nlm.nih.gov/nuccore/539332067). All other relevant data that support the findings of this study are available from the corresponding authors upon reasonable request.

Field-specific reporting

Please select the one below that is the best fit for your research. If you are not sure, read the appropriate sections before making your selection.

- [x] Life sciences
- [ ] Behavioural & social sciences
- [ ] Ecological, evolutionary & environmental sciences

For a reference copy of the document with all sections, see nature.com/documents/nr-reporting-summary-flat.pdf

Life sciences study design

All studies must disclose on these points even when the disclosure is negative.

Sample size

The sample size was based on previous prevention studies in the macaque model, which demonstrate that n=6 per group is sufficient for detecting significant differences in rates of acquisition of infection (Dobard et al., Journal of Infectious Diseases 2020).

For the in vitro assays involving human samples, no sample size calculation was performed as these studies were proof of concept experiments expected to yield binary results, i.e. Leronlimab will/will not prevent replication of CCR5-utilizing strains of HIV or macaques will/will not express similar number of CCR5 molecules. The samples sizes were deemed sufficient after the binary outcome was achieved.

Data exclusions

No data were excluded from the analyses.

Replication

The macaque PrEP study was not run all at once. Instead, it was performed as two separate smaller studies. Any experiments associated with the PrEP study were performed independently for a minimum of two times due to having two separate smaller studies that spanned a period of two years. Similar results were obtained in both smaller studies.

In vitro infection assays, with CCR5 delta 32 human cell ((n=1) were replicated twice in independent runs, while CCR5 WT human cells and macaque cells (n=3 per species) with one replicate each. Assays were not ran all at once but in at least two independent runs.

Viral extraction and preparation for env sequencing were performed in independent experiments but were all combined for a single sequencing run.

Randomization

Animals were first balanced between the experimental groups based on gender. Once gender was balanced, then animals were stratified into groups by weight.

Cells from non-animal experiments were allocated at random, with at least three animals testing each variables. Data was presented as mean values throughout the study. Cells from at least 10 different NHP animal were used in in-vitro studies in this paper.

Blinding

The investigators running and analyzing the assays to measure SHIV plasma and cell-associated viral loads were blinded to the treatment of the study animals during the entirety of the study. The investigators running the ICS assays to assess for SHIV-specific T cell immunity were blinded to the treatment of study animals during the entirety of the study. The investigators running and analyzing ELISA assays to assess antibody concentration and anti-drug antibody (ADA) development were blinded to the treatment groups. No blinding was done for assays measuring receptor occupancy and lymphocyte staining, nor is it relevant because major readout of study was protection from viral acquisition, which viral quantification assays were performed by different investigators.

Because animals were euthanized at ten-weeks post infection or eight-weeks post lost of RO, which ranged from study week 10-38, some investigators were eventually unblinded due to the lengthy period between euthanasias of individual animals.

Reporting for specific materials, systems and methods

We require information from authors about some types of materials, experimental systems and methods used in many studies. Here, indicate whether each material, system or method listed is relevant to your study. If you are not sure if a list item applies to your research, read the appropriate section before selecting a response.

Materials & experimental systems

n/a	Involved in the study	
	x	Antibodies
x		Eukaryotic cell lines
x		Palaeontology and archaeology
	x	Animals and other organisms
	x	Human research participants
x		Clinical data
x		Dual use research of concern

Methods

n/a	Involved in the study	
x		ChIP-seq
	x	Flow cytometry
x		MRI-based neuroimaging

Antibodies

Antibodies used	The following conjugated antibodies were used in these studies: a) from BD Biosciences, D058-1283 (CD45; PE Cy7; 1:100; cat# 561294), SP34-2 (CD3; Alexa 700; 1:100; cat# 557917), SP34-2 (CD3; PE; 1:20; cat# 552127), LP200 (CD4; PerCP-Cy5.5; 1:40; cat# 552838), RPA-T8 (CD8; PacBlu; 1:40; cat# 558207), SK1 (CD8; TruRed; 1:100; cat# 341051) 3GB (CD16; Alexa 700; 1:100; cat# 560713), 25723.11 (IFN-; APC; 1:100; cat# 502512), 6.7 (TNF-; PE; 1:100; cat# 554513), 3A9 (CCR5; APC; 1:100; cat# 560748), SK1 (CD8; BUV737; 1:50; cat# 612754), L200 (CD4; BUV395; 1:200; cat# 564107), FN50 (CD69; PE-Texas Red; 1:100; cat# 562617), SP34-2 (CD3; Pacific Blue; 1:100; cat# 558124), b) from BioLegend, OKT4 (CD4; APC-Cy7; 1:100; cat# 305612), RPA-T4 (CD4; APC; 1:100; cat# 300537), c) from Beckman Coulter, RMO52 (CD14; PE-Texas Red; 1:40; cat# IM2707U), KC57 (HIV Gag p24; FITC; 1:100; cat# 6604665), d) from Sigma, HP-6025 (IgG4; FITC; 3:100; cat# F9890), and e) from SouthernBiotech, HP6023 (mouse anti-human IgG4 pFc'; HRP; 1:20,000; cat# 9190-05), f) from NHP Reagent Resource, 1B3 (anti-rhesus IgG1/3; HRP; 1:5000). The following unconjugated antibodies were used: a) 55-2F12 (SIV Gag p27), conjugated in-house to FITC using PierceTM FITC Antibody Labeling Kit (ThermoFisher) and used at approximately 1:100 depending on the efficacy of conjugation, b) PA-14 (Leronlimab), conjugated in-house to PacBlu using Pacific BlueTM Antibody labeling Kit (ThermoFisher) and used at approximately 1:80 depending on the efficacy of conjugation, c) anti-idiotype antibody, PA-22 (CytoDyn). Live/dead Fixable Yellow Dead Cell Stain Kit and Near-IR Dead Cell Stain Kit (ThermoFisher) were amine-reactive dyes used at 1:1000 dilution to assess cell viability.
Validation	These antibodies are well validated and the details can be found at the nonhuman primate reagent resource website. Antibodies used for flow cytometry were validated by the manufacturer and titrated on rhesus macaque cells and human cells in-house at Vaccine & Gene Therapy Institute at OHSU (Hansen et al., Nature 2013).

Animals and other organisms

Policy information about studies involving animals; ARRIVE guidelines recommended for reporting animal research

Laboratory animals	Rhesus macaques (Macaca mulatta) were included in this study. Both male and female animals were included as well as animals between 2-16 years old.
Wild animals	The study did not involved wild animals.
Field-collected samples	The study did not involve field-collected samples.
Ethics oversight	All rhesus macaques (Macaca mulatta) used in this study were housed at the Oregon National Primate Research Center (ONPRC) and utilized for studies under the approval of the Oregon Health and Science University (OHSU) Institutional Animal Care and Use Committee (IACUC). All macaques in this study were managed according to the ONPRC animal care program, which is fully accredited by AAALAC International and is based on the laws, regulations, and guidelines set forth by the United States Department of Agriculture (e.g., the Animal Welfare Act and its regulations, and the Animal Care Policy Manual), Institute for Laboratory Animal Research (e.g., Guide for the Care and Use of Laboratory Animals, 8th edition), and the Public Health Service Policy on Humane Care and Use of Laboratory Animals.

Note that full information on the approval of the study protocol must also be provided in the manuscript.

Human research participants

Policy information about studies involving human research participants

Population characteristics	The study includes research samples from only one individual, confirmed to have a CCR5 delta 32 homozygous immune system. Individual was a 54 year old male Caucasian.
Recruitment	The individual was aware of the study aims to recapitulate their CCR5 delta 32 homozygous immune system and thus volunteered to partake in the study. The individual was selected as the only individual known cured of HIV due to a stem cell transplant from a CCR5 delta 32 homozygous donor. Thus, it was previously established that T cells from this individual are resistant to CCR5-utilizing HIV strains.
Ethics oversight	The apheresis was approved by the Research and Institutional Review Committee (RIRC) of the Queens Medical Center approval number RA-2014-307 of the H026 Study protocol at the University of Hawaii.

Note that full information on the approval of the study protocol must also be provided in the manuscript.

Flow Cytometry

Plots

Confirm that:

- [x] The axis labels state the marker and fluorochrome used (e.g. CD4-FITC).
- [x] The axis scales are clearly visible. Include numbers along axes only for bottom left plot of group (a 'group' is an analysis of identical markers).
- [x] All plots are contour plots with outliers or pseudocolor plots.
- [x] A numerical value for number of cells or percentage (with statistics) is provided.

Methodology

Sample preparation	The sample preparation is described in sufficient detail to fully recapitulate the process in the supplementary methods under the following sections: HIV and SHIV in vitro infection assays, quantification of CCR5 expression levels, leronlimab CCR5 receptor occupancy, and processing of blood and tissue.
Instrument	BD LSR II Flow Cytometer
Software	Collected using BD FACSDiva v8.01 and analyzed using Flow Jo (Tree Star, Inc.), version 10
Cell population abundance	Not applicable as to cell sorting was performed.
Gating strategy	Gating hierarchies for the spreading assay have been previously published and described in the following:<br><br>Sacha JB, Chung C, Rakasz EG, Spencer SP, Jonas AK, Bean AT, Lee W, Burwitz B, Stephany J, Loffredo JT, Allison DB, Adnan A, Hoji A, Wilson N, Friedrich TC, Lifson JD, Yang OO, Watkins DI. Gag-specific CD8+ T lymphocytes recognize infected cells before AIDS-virus integration and protein expression. Journal of Immunology. 2007, 178:2746-54.<br><br>Gating strategies for macaque immune system subsets including memory T cells and monocytes have been previously described and published in the following:<br><br>Burwitz BJ, Reed JS, Hammond KB, Ericson A, Richter Y, Golomb G, and Sacha JB. Liposomal alendronate depletion of monocytes and macrophages in the nonhuman primate model of human disease. Journal of Leukocyte Biology. 2014 Sep;96 (3):491-501.<br><br>Burwitz BJ, Wu HL, Abdulhaqq S, Shriver-Munsch C, Swanson T, Legasse AW, Hammond KB, Junell SL, Reed J, Bimber BN, Greene JM, Webb GM, Northrup M, Laub W, Kievet P, MacAllister R, Axthelm MK, Ducore R, Lewis A, Colgin LMA, Hobbs T, Martin LD, Thomas CR, Panoskaltsis-Mortari A, Meyers G, Stanton JJ, Maziarz RT, Sacha JB. Allogeneic Stem Cell Transplantation in fully MHC-matched Mauritian Cynomolgus Macaques Recapitulates Diverse Human Clinical Outcomes. Nature Communications. 2017 Nov 10;8(1):1418.<br><br>Gating strategies for CCR5 expression was based on gates set using a FMO control for both CCR5-specific antibodies.

- [x] Tick this box to confirm that a figure exemplifying the gating strategy is provided in the Supplementary Information.
